# Integrin-Mediated Cell-Matrix Interaction in Physiological and Pathological Blood Vessel Formation

**DOI:** 10.1155/2012/125278

**Published:** 2011-09-18

**Authors:** Stephan Niland, Johannes A. Eble

**Affiliations:** Center for Molecular Medicine, Department of Vascular Matrix Biology, Excellence Cluster Cardio-Pulmonary System, J. W. Goethe University Hospital, Theodor-Stern-Kai 7, Building 9 b, 60590 Frankfurt, Germany

## Abstract

Physiological as well as pathological blood vessel formation are fundamentally dependent on cell-matrix interaction. Integrins, a family of major cell adhesion receptors, play a pivotal role in development, maintenance, and remodeling of the vasculature. Cell migration, invasion, and remodeling of the extracellular matrix (ECM) are integrin-regulated processes, and the expression of certain integrins also correlates with tumor progression. Recent advances in the understanding of how integrins are involved in the regulation of blood vessel formation and remodeling during tumor progression are highlighted. The increasing knowledge of integrin function at the molecular level, together with the growing repertoire of integrin inhibitors which allow their selective pharmacological manipulation, makes integrins suited as potential diagnostic markers and therapeutic targets.

## 1. Introduction

Invasive cancer is among the leading causes of death worldwide, and rates are still increasing, due to ageing and changes in lifestyle [1]. Cancer is a collective term for many diseases, rather than a single disease, with the common characteristic that tissue growth goes haywire [2]. Patients who have undergone cancer treatment show an increased risk of developing a second tumor, mainly due to the same risk factors that were responsible for the first tumor but also in part due to the treatment of the first tumor with mutagenic chemotherapeutics or radiation [3]. Therefore, new strategies for cancer treatment with as little as possible adverse side effects are needed that effectively eradicate the primary tumor and also do not increase the risk of recurrence. 

A tumor initially grows without any connection to the vasculature until it reaches a critical size of about two mm in diameter. Then it remains in a dormant state, in which proliferation and apoptosis due to lack of oxygen, are in a dynamic equilibrium unless it develops in a well-vascularized region or is able to recruit its own vasculature. Hanahan and Weinberg have proposed six hallmarks of cancer, one of them being the induction of angiogenesis [4, 5]. For further growth, the tumor needs to hook up to the vascular system by forming neovessels.

During tumor progression, an angiogenic switch is activated causing a continuous neovessel formation emanating from the normally quiescent vasculature, which sustains tumor growth [6]. This process called tumor angiogenesis is a collective term that is generally used for all types of tumor neovascularization. In addition to vessel co-option and to endothelial cell (EC) sprouting, tumor vessels can also develop by intussusceptive or glomerular angiogenesis, or, in a way of vascular mimicry, even tumor cells themselves can form vessel-like hollow structures. These types of vessel formation can occur in parallel, and also gradual transitions are possible. Vessel formation by the latter types requires less energy than sprouting angiogenesis, is thus carried out faster, and usually can be observed in, for example, gliosarcoma multiforme, melanoma, and breast and colon cancer [7].

For neovessel formation, ECs need to migrate into a previously avascular region and to extensively remodel the extracellular matrix (ECM). In this process, integrins, which are cell adhesion receptors for various ECM proteins and immunoglobulin superfamily molecules, are the most important matrix receptors [8, 9]. Therefore, integrins are appealing targets for cancer therapy using a variety of integrin-specific antagonists, ranging from endogenous antagonists over humanized or chimeric antibodies to peptides and small nonpeptidic compounds [10–12].

In this paper, based on the general assembly of blood vessels, the specific organization of tumor vasculature will be described, as well as the dynamic sequence of events by which a tumor gains access to the body's vasculature. In this context, the role of integrins and possibilities of their pharmacological manipulation are explored.

## 2. The Static Picture: The Extracellular Matrix of Blood Vessels

The tissue's ECM is a structure-shaping molecular scaffold and also a repository for cytokines and other growth factors [13]. Cells embedded in this matrix need to be supplied with oxygen and nutrients, signaling molecules need to be received and emitted, and metabolic waste products need to be disposed of. These tasks are optimally fulfilled by the cardiovascular system with its intricate and dynamic network of blood vessels. Depending on their functions, different types of blood vessels show special histological and molecular adaptations. The heart, as a double-acting pump, drives the blood circulation within the vasculature via the aorta through arteries and arterioles into capillaries, from where the blood flows back through venules and veins. Due to the prevailing pressure conditions, the body fluid is forced through the vessel wall to form the lymph, which then is drained by lymph vessels back to the blood circulation. Additionally, the vasculature serves as “highway” system for leukocytes to patrol the body during immunological surveillance and to quickly reach sites of inflammation. The vascular wall is capable of self-sealing upon smaller injuries, and leukocytes are able to penetrate the blood vessel wall in a complex interplay without any obvious vessel leakage. Pathologically, tumor cells capitalize the blood vessel system to disseminate from a primary tumor and to colonize distant organs where they develop metastases. 

### 2.1. General Organization of the Vessel Wall

Histologically, the walls of blood vessels comprise three concentric layers, that is, tunica intima, tunica media, and tunica adventitia [14], which are separated by two sheet-like structures of ECM proteins. The membrana limitans interna and externa establish a border between tunica media and tunica interna and adventitia, respectively. These ECM sheaths tightly connect the cell layers of the vessel wall to form a functional unit, which becomes evident when too weak cell-matrix interactions lead to life-threatening aneurysms. 

The tunica intima comprises a single layer of squamous ECs and lines the inner surface of all blood vessels. The tunica media, which is usually the thickest layer in arteries, is composed of mural cells, which are smooth muscle cells in larger blood vessels and pericytes in capillaries. The tunica adventitia finally interconnects the blood vessel with the surrounding connective tissue, and it is usually most prominent in veins.

In different vessel types, that is, arteries, arterioles, capillaries, venules, and veins, this general blueprint is modified corresponding to the respective functional requirements. For example, endothelia, which are continuous in most instances, can become fenestrated, as in exocrine or endocrine gland tissues, or even discontinuous, as in liver, spleen, or bone marrow, in order to facilitate the exchange of hormones or metabolites. Elastic and muscular arteries illustrate other examples for a modification of this general blueprint. In order to even the pulsatile blood flow coming from the heart, the proteins elastin and fibrillin are abundant in the tunica media ECM of elastic arteries, which is the direct cause for the vessel wall's elastic properties. Muscular arteries possess numerous concentric sheaths of smooth muscle cells. By means of vasoconstriction and vasodilation, they can distribute and direct the blood to different organs.

### 2.2. Extracellular Matrix in the Vessel Wall

The ECM of blood vessels together with their resident cells contributes to essentially all physiological functions of blood vessels and has been reviewed recently [15].

The subendothelial basement membrane (BM) compartmentalizes the vessel's single-layered endothelium from the vascular connective tissue. The molecular architecture of BMs has recently been reviewed [16–18]. Fibronectin, incorporated between endothelial and perivascular cells, is essential for blood vessel morphogenesis [19]. The presence of von Willebrand factor (vWF) is characteristic for the subendothelial BM, where also other BM proteins, such as the network-forming collagens IV and XVIII can be found, together with laminins, nidogens, and perlecan. Thirteen different collagens are present in the vascular wall [20, 21]. The network-forming collagen IV [22] plays a key role for the mechanical stability of the BM [23], which, especially in arterial regions of the circulatory system, has to withstand a considerable blood pressure.

In the tunica media of elastic and muscular arteries, covalently crosslinked supramolecular aggregates of elastin form concentric lamellae and fibers in a proportion of up to 50% of the vessel's dry weight and confer resilience to pulsatile blood flow [24–26]. Regions of the ECM that consist mostly of elastin are confined by EMILINs, that is, homotrimeric elastin microfibril interphase-located proteins [27]. Anchored to microfibrillar bridges of fibrillin-1 and fibulin-5 between these concentric elastin lamellae, vascular smooth muscle cells (VSMCs) are sandwiched in a fishbone-like pattern and thus can effectively regulate the vessel's caliber [25, 28–31]. Dependent on the vessel type, distinct fibulins are involved in the assembly of the ECM. While fibulin-1 is widespread and occurs in the BMs of all blood vessels, heart valves and septa, fibulin-3, and fibulin-4 occur in the walls of capillaries and larger blood vessels [32]. The innermost and outermost elastic lamellae are referred to as membrana limitans interna and membrana limitans externa, respectively. Between the elastic lamellae, type I and III collagens are deposited that bear tensile forces exerted on the vessels and limit their elastic dilatability. In contrast, in the interstitial connective tissue between the subendothelial membrane and the membrana limitans interna, type VI and type VIII collagens are found [21, 33]. The connection of the membrana limitans interna to the subendothelial BM by type XVIII collagen is assumed [34]. Also type XVI collagen, which is produced by VSMCs and found close to both elastic microfibrils and fibrillar type I and type III collagens, may contribute to the connection between the elastic and collagenous phases of the ECM [35, 36], especially, as type XVI collagen contains a binding site for the major collagen receptor on VSMCs, integrin *α1β1* [37, 38].

The ECM of the tunica media is synthesized by VSMCs, which are all encapsulated by an (incomplete) BM containing the usual BM proteins, type IV collagen and laminins [33, 39]. Depending on microenvironmental cues, VSMCs can reversibly acquire distinct phenotypes, which can be characterized as either (i) contractile and differentiated or (ii) secretory, migratory, and less differentiated [37, 39]. Under physiological conditions, the contractile phenotype prevails, at which the VSMCs transduce forces on the pericellular matrix especially by the collagen-binding integrin *α1β1*, by the laminin-binding integrin *α7β1* and by dystroglycan [37]. In contrast, in the secretory, proliferatory, and migratory phenotype, the integrin equipment of the VSMCs predominantly consists of the fibronectin receptor, *α5β1*, and the integrins *α4β1* and *α9β1*. Consistently, in the proximity of secretory VSMCs, the fibronectin splice variants V (IIICS) and EIIIA with binding sites for the integrins *α4β1*, *α5β1*, and *α9β1* are abundant [39]. In capillaries, scattered pericytes, each encapsulated by an own BM, stabilize the endothelium and its subendothelial BM [40–42].

The fibroelastic connective tissue of the tunica adventitia connects the blood vessel with the perivascular connective tissue. It is rich in versican, a glycoprotein, which can interact with fibrillin-1 [43], fibulin-1 [44], and fibulin-2 [45], as well as with other ECM molecules.

### 2.3. Receptors for ECM Molecules

To interact with their microenvironment and to spatiotemporally regulate their differentiation state, morphology, metabolism, and survival, cells are equipped with a variety of receptors for all the ECM molecules [13]. Integrins are the largest family of these receptors, and they mediate adhesion to collagens, laminins, and fibronectin. In addition, there are other receptors and coreceptors, such as the syndecans [46].

Binding to a wide variety of different ECM molecules and transmitting signals bi-directionally in an outside-in and inside-out manner, integrins constitute functional hubs, which, according to an interesting concept in network theory and systems biology, integrate networks of angiogenic signaling cues that orchestrate the behavior of ECs and VSMCs during angiogenesis [47, 48]. Thus, therapeutically targeting integrins as the operationally important circuit-integrating hubs rather than single pathways of the complex system may result in a more pronounced inhibition of angiogenesis [47].

ECs express the vitronectin receptors *α*v*β3* and *α*v*β*5; moreover, on ECs and pericytes the following integrins are expressed: the collagen receptors *α*1*β1* and *α2* *β1*, the laminin receptors *α3* *β1*, *α3β6*, and *α6β4*, the osteopontin receptor *α9β1*, and the fibronectin receptors *α4β1* and *α5β1* [49]. Pericytes additionally express the laminin receptor *α7β1*, and the osteopontin receptor *α* *8* *β1*, and integrin *α*v*β3* is also expressed on glial cells [49].

As EC-derived tumors, angiosarcomas express the integrins *α1β1*, *α2* *β1*,*α3* *β1*,*α5β1*, and *α6* *β1*, and in benign and malignant mesenchymal tumors as well as in the desmoplastic stroma of carcinomas, integrins *α1β1* and *α5β1* are widely distributed [50]. Integrins *α1β1* and *α2* *β1* bind to the same ligand in the ECM and are VEGF-dependently upregulated on migrating ECs, and antagonists against both integrins inhibit VEGF-mediated angiogenesis without affecting the existing vasculature [51, 52]. Therefore, and against the background of gene ablation studies, they are believed to differentially regulate angiogenesis [49]. Important coreceptors for integrin *α2* *β1* are the syndecans-1 and -4, which weaken the invasiveness of tumor cells into a collagenous matrix [53].

Cells bind to fibronectin and vitronectin preferentially via the RGD-dependent integrins *α*v*β3* and *α5β1* [54]. Fibronectin can also be bound by the leukocyte-specific integrins *α4β1* and *α4β7* [55]. Cell-fibronectin interactions are modulated by proteoglycans, glycoproteins of the ECM, and the coreceptors syndecans [56].

Integrin *α*v*β*3 was identified as a marker for angiogenic vascular tissue [57]. In contrast to quiescent ECs, integrin *α*v*β*3 is highly expressed on activated ECs during tumor angiogenesis, as well as on some tumor cells [58, 59]. In the tumor microenvironment, angiogenic ECs can interact due to their increased levels of the integrins *α*v*β*3 and *α*v*β*5 with provisional matrix proteins, such as vitronectin, fibrinogen, vWF, osteopontin, and fibronectin. Also, partially proteolyzed collagen in the tumor exposes RGD sites and is a further ligand for integrin *α*v*β*3 [60]. Thus, the ECM of the tumor microenvironment both provides survival signals and facilitates invasion. Integrin-*α*v*β*3-mediated adhesion to platelets protects malignant cells from clearance through the immune system, and moreover, *α*v*β*3 integrin also helps tumor cells to adhere to the vessel endothelium and to spread into adjacent tissues [61].

The pharmacological inhibition of integrin-*α*v*β*3-mediated cell-matrix interaction impedes tumor angiogenesis and growth [62], as does a replacement of the *β*3 subunit with a mutated nonphosphorylatable subunit in a murine model [63], which provides evidence for a proangiogenic role of integrin *α*v*β*3, in contrast to integrin *α*v*β*5, which does not seem to play an essential role in angiogenesis [64]. Interestingly, the analysis of *α*v-knock-out mice revealed that, despite being embryonic or perinatally lethal, the vascular endothelium was not impaired in the absence of the *α*v subunit, whereas the primary cause of death was brain hemorrhage [65–67]. Also endothelial Tie-2-specific knockout of the *α*v subunit did not result in any vascular or angiogenesis defect [67]. Moreover, in an integrin subunit *β*3- and also *β*5-deficient mouse model, pathologic angiogenesis and tumor growth are increased [68]. A possible cause for these seemingly contradictory phenomena could be a relief of a transdominant inhibition by *α*v*β3* on other integrins or other molecules, which would enhance their proangiogenic function [69, 70]. Likewise, there could be a compensatory role of other integrins with overlapping function [49]. Moreover, inhibition could also stabilize the integrin *α*v*β3* in its unligated conformation and thus induce apoptosis by triggering an integrin-mediated death program [71].

Integrin *α*v*β8* is important for vascular development in the embryonic brain and in the yolk sac [72]. It is expressed on astrocytes but not on ECs or pericytes, nevertheless plays an important role in angiogenesis, as it binds in addition to several ECM proteins also to the latency-associated peptide (LAP) of TGF*β*1, which in cooperation with the membrane-type metalloproteinase MT1-MMP/MMP14 results in activation of TGF*β* and triggering of its downstream signal cascades [73–75].

Collagen IV, an essential component of BMs, is bound by integrin *α1β1*, which is expressed on mesenchymal cells and can also bind to other collagens [76, 77]. Further collagen-binding integrins are *α2* *β1*, the main receptor for fibrillar collagens, which is expressed on epithelial and some mesenchymal cells as well as on thrombocytes [78], *α1* *0* *β1* in cartilage [79], and *α1* *1* *β1*, a key receptor for fibrillar collagen on fibroblasts [80]. The integrins *α1β1* and *α2* *β1* are involved in the regulation of collagen and MMP synthesis and thus of special importance for ECM turnover [81–83]. Discoidin domain receptors DDR1 on epithelial cells and DDR2 on mesenchymal cells are further collagen receptors with tyrosine kinase function and are relevant for cancer [84]. Other collagen receptors are glycoprotein GPIV on platelets [85], the leukocyte-associated immunoglobulin-like receptor LAIR-1/CD305 [86], and the urokinase-type plasminogen activator receptor-associated protein uPARAP/Endo180, which is involved in matrix turnover during malignancy [87].

Laminin, as a further integral component of BMs, is bound by the integrins *α3* *β1*, *α6* *β1*, *α6β4*, and *α7β1* [88–91] and also by *α*-dystroglycan [92, 93] and by the 67 kDa laminin receptor 67LR [94]. 67LR is increased in various tumors and correlates with their metastatic potential [95, 96]. The different laminin receptors may also act cooperatively in laminin binding, for example, laminin-binding *β1* integrins and 67LR [97] or integrin *α6β4* and syndecan 1 [98].

Integrin *α3* *β1*, which in the vascular wall binds to laminins-411 (laminin 8) and-511 (laminin 10), thrombospondin (TSP), TIMP2, tetraspanin CD151, and to the C-terminal domain of the collagen IV *α3* chain, is controversially ascribed either a positive or a negative role in angiogenesis (cf. [99]).

There is controversy whether the hemidesmosomal integrin *α6β4*, which is expressed on a subset of ECs [100] and on tumor ECs [101], aggravates pathological angiogenesis [101] or whether it is a negative regulator of angiogenesis that is downregulated at its onset [102].

Thus, many molecules of the ECM scaffold, for example, laminins, collagens, fibronectin, and vitronectin, are ligands for integrins that link the cell's cytoskeleton to the ECM. Loss of this matrix-integrin contact triggers apoptotic cell death [103]. Picking up signals from the cell's microenvironment, integrins functionally sense, interpret, and distribute information, which allows the cell to modulate its proliferation, differentiation, migration, and shape [104]. The modulatory and regulating function of integrins is emphasized by direct interaction with a multitude of proteins, such as MMPs, uPA/uPAR, tissue inhibitor of matrixmetalloproteinase-2 (TIMP-2), vWF, TSP-1, osteopontin, syndecan-1, insulin-receptor substrate-1 (IRS-1), cytohesin-1, integrin cytoplasmic domain-associated protein-1 (ICAP-1), integrin-linked kinase (ILK), calcium- and integrin-binding protein (CIB), *β3*-endotoxin, talin, actinin, tensin, nischarin, and the Ras-related protein Rab 25 [9].

The subendothelial BM of the tunica intima serves as a mechanical support to which ECs are anchored by various adhesion molecules, especially integrins [46, 105–108]. Additionally, the subendothelial BM provides microenvironmental information that regulate the metabolic activity of attached ECs, such as their production of leukocyte adhesion molecules [107] or antithrombotic prostacyclins [109], as well as other properties, for example, the tightness of intercellular contacts [108]. Therefore, angiogenesis is regulated not least by integrins which are adhesion receptors for matricellular proteins, ECM proteins, and immunoglobulin superfamily molecules, on nearly all cells including ECs [8, 58].

In addition to their mechanical function [110], integrins also assist growth factor receptors and play important roles in signaling processes, in particular as soluble growth factors, and other signaling molecules are bound by integrins as well [111]. For example, the proangiogenic VEGF-A_165_ is bound by integrins *α*v*β3* and a*3* *β1* [112] and also by the tenascin-C- and osteopontin-receptor integrin *α9β1* [113]. The latter integrin, furthermore, binds the lymphangiogenic growth factors VEGF-C and VEGF-D [114]. Angiopoietins-1 and -2 are bound by integrin *α5β1* [115]. Integrin *α6* *β1* is a receptor for the proangiogenic CCN-family member CYR61, and is involved in *in vivo* in tube formation [116, 117]. The fibronectin receptor integrin *α*v*β3*, which is the best-studied integrin in relation to angiogenesis and is upregulated during wound healing and retinal vascularization and especially on tumor blood vessels, also binds to fibroblast growth factor FGF-1 [118]. Semaphorin 7A binding is also reported for the collagen receptor integrin *α1β1* [119].

Stimulated by PDGF, vascular smooth muscle cells express the laminin receptor integrin *α7β1*, which plays an important role in recruitment and differentiation of VSMCs [120, 121].

Integrin *α9β1* is not only involved in lymphangiogenesis [114] but also plays a role in EC adhesion [122]. While binding of TSP-1 to integrin *α9β1* promotes angiogenesis [123], VEGF-A is another ligand of integrin *α9β1* [113].

### 2.4. Vascular-Relevant Integrin-Deficient Mouse Models

The crucial involvement of integrins in EC biology has been elucidated substantially by the examination of genetic knock-out studies [124]. By ablation of the respective genes, the EC integrins *α*1*β1*, *α2* *β1*, *α4β1*, *α5β1*, *α6* *β1*, *α6β4*, *α*9*β*1, *α*v*β*3, and *α*v*β*5 and also the VSMC integrin *α*7*β*1 and the glial cell integrin *α*v*β*8 have been implicated in regulation of cell growth, survival, and migration during angiogenesis (for recent reviews of the findings from knock-out mice cf. [8, 10]). However, due to redundancy and compensatory mechanisms, the interpretation of knock-out results is often difficult.

Itgb1−/− mice die at E5.5 before they start to develop their vasculature [125, 126]. Mice with a conditional knockout in Tie-2-positive ECs survive until E9.5–E10.5, and they are capable of vasculogenesis, but their angiogenesis is disturbed showing defects in sprouting and branching [127–129]. Another endothelial-specific knockout of the integrin *β1* subunit is mediated via VE-cadherin-Cre recombinase and becomes manifest later in embryogenesis resulting in lethality between E13.5 and E17.5 [130]. In this mouse model, loss of *β1* integrin leads to a decreased expression of the cell polarity gene PAR3 and thus to disruption of EC polarity and lumen formation [130].

Itga1−/− mice, deficient for the collagen-binding integrin *α1β1*, show a normal vascular development and a reduced tumor angiogenesis in adulthood, which has been attributed to increased MMP activity [131], while *α2* *β1*-deficient Itga2−/− mice show an enhanced tumor angiogenesis in adulthood, but an otherwise normal vascular development [131, 132], and integrin *α2* *β1* is involved in the PlGF-dependent regulation of VEGFR-1 [132]. Although integrin *α1β1* and *α2* *β1* bind to the same ligand in the ECM, their differential knockout results in opposing effects on angiogenesis, suggesting a regulatory role for this pair of integrins.

Da Silva and coworkers generated EC-specific conditional *α3* integrin knock-out mice and showed that these mice, in contrast to a global ablation, are viable and fertile but display enhanced tumor growth, elevated hypoxia-induced retinal angiogenesis and tumor angiogenesis, and increased VEGF-mediated neovascularization [99]. The authors also could show that *α3* *β1* is a positive regulator of EC-derived VEGF, which again represses VEGFR2 expression. Their data demonstrated that endothelial *α3* *β1* negatively regulates pathological angiogenesis and implicated an unexpected role for low levels of EC-derived VEGF as an activator of neovascularization.

Itga4−/− mice, deficient for fibronectin- and VCAM1-binding integrin *α4β1*, are embryonic lethal with 50% dying at E9.5–10.5 due to failure of chorion-allantois fusion and 50% dying at E11.5 due to cardiovascular defects [55].

Mice, which by ablation of Itga5 are deficient for the fibronectin receptor integrin *α5β1*, show normal vasculogenesis but no angiogenesis, which results in embryonic lethality at E10-11 due to defects in posterior somites, yolk sac, and embryonic vessels [133, 134]. This demonstrates the requirement of the integrin *α5* subunit during embryonic development of early blood vessels and other tissues. Accordingly, integrin *α5β1*, which is poorly expressed on normal quiescent ECs, is markedly upregulated during tumor angiogenesis [135].

Among the laminin-binding integrins, integrin *α6* is not essentially required for vascular development, although *α6*-deficiency is lethal with skin blistering defects resembling epidermolysis bullosa [136]. In line with the *α6* knockout mice, Itgb4−/− mice, lacking a functional laminin-binding integrin *α6β4* by deletion of its signaling domain, show normal vascular development, although with reduced angiogenesis [101], but die of severe skin defects [100]. In neovascularization, the endothelial expression of integrin *α6* *β1* is downregulated [102]. While it is not required for EC proliferation and survival, it promotes tumor angiogenesis [101]. In contrast, genetic ablation of *α7β1*, which is expressed on VSMCs but not on ECs, leads to incomplete cerebral vascularization and hemorrhage and also to placental vascular defects, which results in partial embryonic lethality and demonstrates that integrin *α7β1* is important for recruitment and survival of VSMCs [121, 137].

Deletion of Itga8 resulting in lack of integrin *α* *8* *β1*, a receptor for fibronectin and tenascin, results in partial embryonic lethality, but no defects in vascular development (Müller and Reichardt, cited in [138]). 

Itga9−/− mice lacking integrin *α9β1*, which is the receptor for tenascin-C, osteopontin, VCAM-1, and also for VEGF-A, -C, and -D [113, 114], have defects in large lymphatic vessels and die postnatally at P8-12 from a bilateral chylothorax [139].

Ablation of Itgav, resulting in simultaneous loss of the two integrins *α*v*β5*, a receptor for vitronectin, osteopontin, and Del-1 (developmental locus 1), and *α*v*β3*, a receptor for a variety of ECM proteins, such as fibronectin, vitronectin, laminins, fibrinogen, fibrin, TSP, tenascin-C, vWF, denatured collagen, osteopontin, MMP-2, Del-1, bone sialoprotein, FGF-2, thrombin, and CCN1 (cystein-rich protein 61), leads to 80% embryonic lethality at E9.5, and the other 20% die at P0 with brain hemorrhage [65]. On the other hand, Itgb3−/− mice, which are just integrin-*α*v*β3* deficient, show 50% embryonic and early postnatal lethality and an enhanced angiogenesis in surviving adult animals, indicating that this integrin is not strictly required for vascular development [140]. Surprisingly, animals with an intact but nonfunctional *β3* integrin subunit develop normally but show defects in angiogenesis in adulthood [63]. In contrast, Itgb5−/− animals lacking integrin *α*v*β5* develop normally and angiogenesis is not significantly affected, indicating that this integrin is not mandatory for vascular development [64]. Integrins *β*3 and *β*5 doubly deficient mice show enhanced tumor growth and angiogenesis. This strongly suggests that these integrins are not required for vascular development or for pathological angiogenesis, pointing out that the mode of action of *α*v*β3* antagonists and antiangiogenic therapeutics is still insufficiently understood [68]. Ablation of Itgb8 leads to the loss of integrin *α*v*β8* on glial cells and thus to disrupted blood vessel formation in the brain, thereby demonstrating that this integrin is mandatory for brain's blood vessel development [72]. Moreover, the phenotype of *β8*-deficient mice resembles that of *α*v-deficient mice, which provides evidence that most defects in *α*v-deficient mice are due to the loss of integrin *α*v*β8* [72].

### 2.5. Integrin Structure

The family of integrins contains 24 structurally related N-glycosylated heterodimeric proteins assembled noncovalently from 18 *α*-subunits and eight *β*-subunits. Each subunit comprises a large extracellular domain, a single transmembrane domain, and with the exception of the *β*4 integrin subunit, a short noncatalytic cytoplasmic tail [141]. Integrins are of special importance as they mediate cell matrix crosstalk via both outside-in and inside-out signaling [54, 142]. Moreover, the 24 different integrins possess promiscuous and redundant ligand specificities, which is of importance when distinct signals are to be transduced or when in a particular context a defined cellular response is elicited, as is discussed by Rüegg and Alghisi [11].

Integrin structure and function have been studied in detail at the molecular level [143, 144]. The extracellular headpiece is formed by a disk-like propeller domain of the *α* subunit and globular domains of the *β* subunit [145, 146]. The joint globular head harbors the ligand-binding site [146, 147]. The crystal structure of the integrin-*α*v*β3*-binding site with an inserted RGD ligand [148] helped to map functional amino acid residues on other integrins [149]. Recently, the binding pocket of integrin *α5β1* has been mapped by swapping regions of zebrafish and human *α5* subunit in a gain-of-function approach [150].

### 2.6. Integrin Signaling

**Figure 1:**
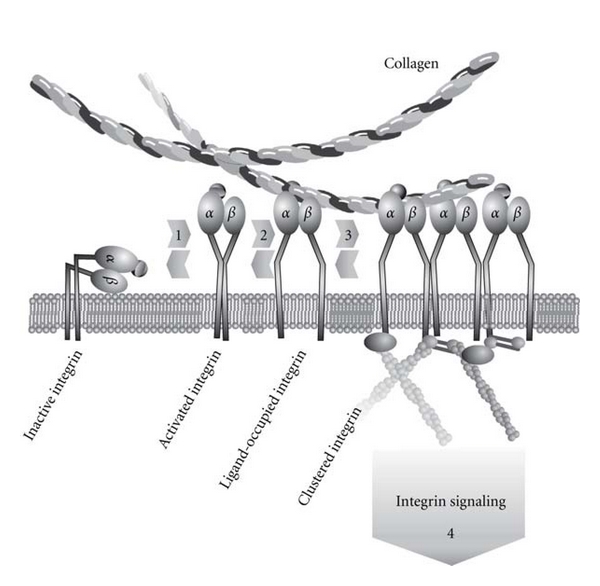
Integrin activation. Integrins are a family of heterodimeric transmembrane adhesion receptors that bidirectionally relay signals with the extracellular matrix (ECM) and also with other cells. When activated, a conformational change increases the affinity, and clustering increases the avidity towards the ligand. (1) By inside-out signaling, integrins can reversibly undergo a conformational change from a bent inactive to an upright activated conformation with intermediate ligand affinity, at which the cytoplasmic domains are still close together. (2) Upon ligand binding, the integrin adopts a high-affinity conformation with a concomitant parting of the legs and a separation of the cytosolic *α*- and *β*-tails that unlocks docking sites for cytosolic molecules. (3) Clustering of ligand-occupied and activated integrins establishes a mechanical link between ECM and cytoskeleton and leads to the recruitment of scaffolding molecules and kinases. (4) The assembly of focal adhesions triggers intracellular signaling cascades. Details can be found in the text.

Depending on their activity, integrins adopt distinct conformations (Figure 1). In the inactive resting conformation, the headpiece of the heterodimer bends towards the plasma membrane, and the transmembrane domains of the *α* and *β* subunits are associated [146]. Upon ligand binding, the previously bent integrin ectodomain adopts an activated upright conformation [106, 151]. This conformational change is conveyed through the transmembrane domains towards the cytoplasmic tails [54, 105, 152], where cytoskeletal proteins and signaling molecules relay the incoming signal intracellularly [153]. In inside-out signaling, the binding of intracellular molecules, such as talin or kindlins [154, 155], to the cytoplasmic integrin tails leads via a separation of the transmembrane domains [156] to a switch blade-like erection of the extracellular domains [147, 157, 158]. Likewise, in outside-in signaling, ECM ligand binding to the integrin headpiece also induces a conformational change in the hybrid domain and thereby a separation of the integrin subunits' legs [144]. This parting of the legs separates the cytosolic tails and allows binding of cytosolic proteins and thus clustering of integrins and formation of focal adhesion sites (Figure 1).

By clustering into focal adhesions, integrins recruit talin, paxillin, *α*-actinin, tensin, and vinculin and thereby mechanically couple the ECM scaffold to the actin cytoskeleton. Additionally, integrins bind scaffolding molecules, such as p130 CRK/BCAR1, and recruit and activate kinases, such as focal adhesion kinases (FAKs), Src family kinases (SFKs), and integrin-linked kinase (ILK), the latter forming a complex with the adapter molecules parvin and PINCH/LIMS1 [159]. In addition, tetraspanins can recruit integrins to membrane microdomains, thus regulating integrin function [160]. Thereby, the rather unstable nascent adhesions are transformed into focal complexes, focal adhesions, fibrillar adhesions, or podosomes. This clustering of integrins leads to a reorganization of the plasma membrane around the focal adhesion into caveolin-containing lipid rafts, to which also growth factor receptors often localize, and to the assembly of adhesion signaling complexes [161–163]. This allows a regulation of growth factor signals by integrin-mediated caveolae trafficking [164, 165]. In the assembly of such integrin adhesions, up to 156 distinct molecules, amongst other adaptor proteins, kinases, and phosphatases, are involved [48, 163]. Membrane lipid-protein interactions that modulate the homo- or heterotypic association of receptor molecules in the cell surface, or between adjacent cells, have been reviewed recently [166]. From the focal adhesion sites signal pathways diverge that regulate diverse cellular programs, such as adhesion, migration, proliferation, and survival. To provide an overview, integrins generally relay their signals via the FAK, ERK, and NF-*κ*B pathways [153].

In most cases, in mechanosensory signaling FAK, Src, and SH2, domains containing protein tyrosine phosphatase 2 (SHP2) are involved [167]. Upon integrin binding, FAK autophosphorylates and binds to Src, which further phosphorylates FAK and several downstream binding partners, amongst others, JNK and Rho [168–170].

Activated FAK also recruits PI3K, which mediates the activation of AKT and procures integrin-mediated cell survival, and likewise the antiapoptotic AKT can be activated via Ang-1 [171]. Moreover, signals relayed via integrins and Src can be integrated by FAK with growth factor receptor-relayed signals via Ras, MEK, and MAPK [172]. Growth factors can activate Ras signaling independently from integrin-relayed adhesion signals. Nevertheless, MEK1 and Raf1 are important interfaces between integrin-relayed and growth-factor-relayed signaling, because both MEK1 and Raf1 need to be activated via adhesion-mediated activation of Src and FAK in order to activate MAPK [173, 174].

An endothelial-specific ablation of FAK results in impaired blood vessel development and embryonic lethality [175] Downstream of FAK, Src couples integrin-mediated and VEGF-receptor-mediated proangiogenic signaling in ECs [176–178]. However, endostatin can also activate Src via integrin *α5β1* and thereby disassemble actin stress fibers and focal adhesions and thus inhibit cell migration, which is regulated by integrins via the Ras/ERK pathway [179–181]. Important for adhesion and migration of endothelial and VSMCs are also p130Cas and PLC-*γ*, which can interact with FAK [182–185].

PI3K is of pivotal importance for angiogenesis, because its deletion results in embryonic lethality E9.5 to E10.5, when angiogenesis is important for vascular development. PI3K deletion also causes decreased Tie-2 expression and thus creates a phenotype resembling Tie-2 deficiency [186, 187]. Moreover, EC-specific deletion of the PI3K isoform p110*α* impairs angiogenesis [188]. In ECs, adhesion via integrins elicits a survival signal via FAK/PI3K/mTOR/4E-BP1 and Cap-dependent translation [189]. Furthermore, the activation of PI3K by Ras is important for lymphangiogenesis [190].

In addition to a direct activation of ERK, integrins can also activate a Raf/MEK/ERK signaling cascade in ECs [189, 191, 192]. Raf-deficient and MEK-deficient mice have severe vascular defects [193, 194]. Growth-factor-mediated ERK signaling is linked with integrin-mediated signaling via FAK [195]. Integrin-mediated ERK signaling is important for cell proliferation and migration of ECs [191, 196]. Integrin *α1β1* is unique among the collagen-binding integrins because it promotes cell proliferation by activating the Ras-Shc-MAPK pathway, and cell cycle progression is regulated via FAK, Rac, and cyclin D by integrin-mediated adhesion and matrix stiffness [197–199].

Integrins can also activate the NF-*κ*B pathway in ECs and protect them from apoptosis [200–202]. Additionally, NF-*κ*B signaling regulates the expression of cyclooxgenase-2 (COX-2), which again is involved in EC spreading and migration and in the induction of VEGF and FGF-2 [177, 203, 204]. However, inhibition of the NF-*κ*B pathway increases angiogenesis pathologically [205].

Integrins alone are not oncogenic, but some oncogenes may depend on integrin signaling for tumor growth and invasion. For example, integrin-triggered FAK signaling is essential for Ras- and PI3K-mediated oncogenesis [206, 207]. Also the expression of the cancer stem cell marker CD44 is integrin-regulated, and it can be speculated that integrin-relayed signals are needed to maintain a cancer stem cell population [12, 208]. On the other hand, there is evidence that the collagen receptor integrin *α2* *β1* has a tumor-suppressing function [209, 210].

Ligated integrins promote survival, whereas unligated integrins recruit caspase-8 to the plasma membrane and promote apoptosis in a process termed integrin-mediated death [71, 211], which differs from anoikis induced by loss of cell adhesion to the ECM [103, 212]. Loss of caspase-8 confers resistance to integrin-mediated death of tumor cells, and unligated integrin *α*v*β3* promotes the malignancy of such tumors [213, 214]. Cell survival is promoted by integrin ligation-dependent upregulation of BCL2 and FLIP/CFLAR, activation of the PI3K-AKT pathway, NF-*κ*B signaling, and p53 inactivation [176, 202, 215–217]. Survival is also promoted by crosstalk between integrins and growth factor receptors, for example, *α*v*β3* and FGFR or *α*v*β5* and VEGFR2 [195, 218].

In various steps of angiogenesis and tumor progression, crosstalk between integrins and growth factor receptors on tumor cells and also on host cells is important. This crosstalk can consist in either an activation of a latent growth factor, a regulation of common pathways for signaling or internalization and recycling, a collaborative or a direct activation, or also a negative regulation [111]. The outcome of a growth factor signal in a particular context is often determined by a synergistic and reciprocal interaction of integrins with growth factor receptors, such as tyrosine kinase receptors like VEGFRs and Tie-2, Met, and FGFR, and semaphorins regulate integrin function as well [111, 219–221]. A complex of VEGF with the fibronectin heparin II domain increases, upon cell binding via integrin *α5β1* and the signaling via VEGFR2 synergistically [222]. Expression of integrin *α1* *1* *β1* on tumor-associated fibroblasts has a tumor-promoting effect, because it upregulates the expression of insulin-like growth factor 2 (IGF2), which is another example of integrin-regulated growth factor signaling [223].

Beside binding ECM proteins and thus regulating adhesion and migration, integrins can also directly interact with pro- and antiangiogenic factors [221]. Integrin *α5β1* can bind to matrix-bound VEGFR-1 [224]. In addition, integrin *α9β1* can directly interact with VEGF-A, -C, and -D and also with hepatocyte growth factor (HGF) [113, 114, 225]. Moreover, integrin *α3* *β1* and *α*v*β3* bind VEGF-A_165_ and VEGF-A_189_ [112]. FGF is directly bound by integrin *α*v*β3* [226]. Angiopoietins also can directly interact with many integrins [115, 221, 227, 228].

In the context of a hypoxic tumor microenvironment, it is especially interesting that the expression of integrins *α*1*β1* and *α*2*β1* is upregulated by VEGF [51].

## 3. The Dynamic Process: Connection of a Tumor to the Host Vasculature

Angiogenesis is an important step in the metastatic cascade, which not only provides the tumor with nutrients but also is a route for dissemination. An important trigger for this is hypoxia [229].

### 3.1. An Angiogenic Switch Triggers the Angiogenic Cascade

In avascular tissue regions, an oxygen diffusion limit of about 150 *μ*m restricts tumor growth to just a few millimeters in diameter. Thus, in this prevascular phase of tumor dormancy, there is a dynamic equilibrium between proliferation and hypoxia-induced apoptosis [230]. The dormant phase ceases when a tumor recruits its own vasculature by the secretion of angiogenic factors into its environment [231], a process denoted as angiogenic switch [2, 6]. After this angiogenic switch is thrown, the tumor hooks up to the body's vascular system and thus resumes its growth.

In tumor development, the establishment of an angiogenic phenotype is a crucial and general step [232–234]. Depending on tumor type and environment, this induction of new vessel sprouting can occur at different stages of the tumor progression pathway, and it leads to exponential macroscopic tumor growth [2, 4, 6]. In addition, recent data indicate that angiogenesis also contributes to the microscopic premalignant phase of neoplastic progression [5].

Infiltration of bone-marrow-derived monocytes that differentiate into macrophages can trigger this angiogenic switch in spontaneous tumors by releasing both numerous proangiogenic cytokines, for example, VEGF, TNF*α*, IL-8, and bFGF [235, 236] and MMPs (e.g., MMPs-2, -7, and -9) together with elastase and uPA [236]. These matrix-degrading enzymes loosen the avascular ECM for the angiogenic ingrowth of neovessels.

From the multitude of proangiogenic molecules, such as FGF-1 and -2, G-CSF, HGF, IL-8, PD-ECGF, PGE-1 and -2, PlGF-1, and -2, TGF-*α* and -*β*, TNF-*α*, and VEGF-A through E, only the VEGFs and PlGFs are specific for ECs [230]. VEGF-A, which exists in five splice variants, is the most intensively studied one [237]. Mediated by HIF-1, VEGF-A synergizes with FGF-2. VEGF is upregulated under hypoxic and hypoglycemic conditions prevailing within tumor tissue [230].

The role of chemokines in tumor angiogenesis and neovascularization has been reviewed recently [238]. Tumor cells express CCL2/MCP-1 (C-C-motif ligand 2/monocyte chemotactic protein-1), and thus, tumor-associated macrophages (TAMs) are recruited, resulting in an inflammatory response. These TAMs are again a source for angiogenic growth factors, such as, VEGF and FGF-2 [239, 240]. MCP-1 also mediates the recruitment of mural cells in an Ang-1-dependent manner in an *ex vivo* model [241].

Multiple sequential steps are required for angiogenesis to be successful and in all steps of this angiogenic cascade integrins, which mediate interactions of cells with surrounding insoluble ECM proteins, in addition to soluble growth factors, play an important role [15]. In a first step, the BM of an existing vessel is degraded by MMPs that are expressed by ECs, such as MMP-1, MMP-2, MMP-9, and MT1-MMP/MMP14 [242–244], at which MMP-9 is required for tumor vasculogenesis rather than angiogenesis [245]. Subsequently, cell-matrix contact influences the outgrowth of tip cells and the proliferation of stalk cells that thereupon form endothelial tubes [246]. A new BM is assembled by newly synthesized BM proteins. Finally, the newly generated capillaries undergo maturation, pruning, and expansion.

### 3.2. Tumor Vessels Can Arise by Different Types of Vessel Formation

During embryonic morphogenesis, endothelial precursor cells called angioblasts initiate the body's vasculature by forming tubes in a process called vasculogenesis. This is subsequently accompanied by sprouting (angiogenesis) of new vessels from already existing ones. Once morphogenesis is completed, the adult vasculature is largely quiescent, except for transient events, such as wound healing or menorrhea [247]. However, angiogenesis takes place under many pathological conditions, such as atherosclerosis, endometriosis, osteomyelitis, diabetic retinopathy, rheumatoid arthritis, psoriasis, and tumor growth [230]. During tumor progression, the quiescent vasculature becomes permanently activated to sprout new vessels that enable blood supply and thus help sustain tumor growth [5, 6]. Due to its increased metabolic rate, tumor tissue requires blood supply for expansive growth, which is circumstantiated by the observation that tumor cells, which are p53 deficient and thus show a reduced apoptosis rate, die beyond an oxygen diffusion limit in the range of 150 *μ*m [248]. Tumor cells proliferate around the continuously formed neovessels which markedly differ from normal vessels in morphology and molecular composition [219, 249]. Tumor vasculature generally appears highly tortuous, chaotic, and disorganized. The vessels themselves are leaky due to a discontinuous endothelium, a poorly formed BM, and a lack of mural cells. In addition, tumor cells sometimes mimic ECs. This poor quality of tumor-associated blood vessels compromises blood flow, impairs drug delivery, and facilitates tumor cell intravasation leading to hematogenous or lymphatic metastasis. In addition to histological vessel malformations, tumor vessels show an anomalous composition of their ECM, for example, tenascin-C and –W, and the oncofetal fibronectin ED-B splice variants are associated with tumor vessels [250, 251]. ED-B fibronectin is synthesized by neoplastic cells [252]. Melanoma and glioblastoma cells secrete tenascin-C as do cancer-associated fibroblasts (CAF) of most carcinomas [253]. Tenascin-C stimulates angiogenesis in ECs, mediates survival of tumor stem cells, enhances proliferation, invasiveness, and metastasis in tumor cells, and blocks immunosurveillance [250, 253]. Tenascin-W is more strictly associated with tumorigenesis and can be used as a tumor biomarker for breast and colon cancer, because it is undetectable in healthy stroma but overexpressed in the tumor stroma [254, 255].

**Figure 2:**
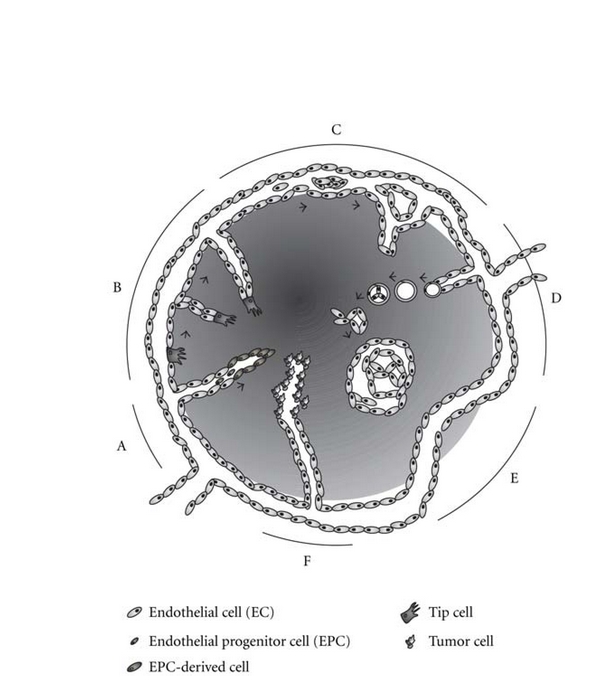
Diverse types of vessel formation. Tumor neovascularization can take place by distinct types of vessel formation, which can proceed simultaneously and also merge seamlessly. (A) Neovessel formation by recruitment of bone-marrow-derived endothelial progenitor cells. (B) Sprouting angiogenesis is initiated by the differentiation of an EC into a migratory but nonproliferating tip cell. (C) Intussusceptive angiogenesis starts with the insertion of a connective tissue pillar into a preexisting vessel, and the vessel is displaced as the pillar extends in size. (D) In glomeruloid angiogenesis, complex vascular aggregates of several closely associated vessels are formed. (E) Vessel co-option is the acquisition of host capillaries by the tumor. (F) In vascular mimicry, tumor cells can partly assume EC function and form vessel-like hollow structures. Arrows denote consecutive stages of vessel formation. Tumor tissue is depicted dark gray. See text for details.

Vascularization mechanisms in cancer have been reviewed recently [256, 257]. New tumor blood vessels can either arise by vessel co-option or be formed by tumor angiogenesis, but there is also evidence for vasculogenesis or recruitment of circulating bone-marrow-derived endothelial progenitor cells that differentiate into ECs [230, 258–260] (Figure 2(A)). Depending on the tumor type, tumor blood vessels build different and characteristic vascular beds, and, according to the function of the vascular bed and the osmotic pressure of the surrounding tissue, endothelia represent highly heterogeneous “vascular addresses” [230]. Tumor vessels constantly change their shape due to persistent growth, and about 30% of the vasculature comprise arteriovenous shunts bypassing capillaries. The concomitant poor perfusion leads to hypoxia of ECs, which consequently synthesize more proangiogenic molecules and thus crank tumor angiogenesis [230].

#### 3.2.1. Endothelial Sprouting

Endothelial sprouting can be triggered by hypoxia, hypoglycemia, and inflammatory or mechanical stimuli, such as blood pressure, and is regulated by many angiogenic growth factors, such as VEGF, and matrix proteases. When neovessels sprout from capillaries, pericytes are selectively lost, and upon receiving an angiogenic stimulus, select ECs differentiate into tip cells that invade the avascular ECM (Figure 2(B)). These tip cells migrate into the ECM following the stimulatory gradient. Behind the tip cells, other ECs begin to proliferate and, as stalk cells, form cord-like structures. These develop into endothelial tubes [130, 261, 262] that subsequently anastomose and thus allow blood flow. Finally, pericytes and smooth muscle cells are recruited, a new BM is synthesized, and the ECs become quiescent again.

The molecular background of capillary sprouting and the key role of VEGF have been reviewed by Carmeliet [231]. Upon a hypoxic stimulus, VEGF is produced, and as a consequence the endothelium's permeability is increased and the BM loosened by the activity of MMPs [243, 263] and the urokinase plasminogen activator system [264]. The MMP inducer EMMPRIN/CD147 also upregulates soluble VEGF isoforms 121 and 165 and VEGFR-2 on ECs and thus promotes sprouting angiogenesis [265]. Integrin *α*v*β3* mediates migration into the fibrin-rich cancer stroma and furthermore can associate with MMP-2, thus enabling ECs to maintain the BM in the sol state and to promote tumor cell invasion [266]. In addition to VEGF, FGF, PDGF, and PlDGF are involved, and Ang-2/Tie-2 signaling regulates the detachment of pericytes. Later, PDGF-BB recruits pericytes and smooth muscle cells to the newly formed EC tube, and TGF-*β*1 and Ang-1/Tie-2 stabilize the EC-mural cell interaction [231].

#### 3.2.2. Intussusceptive Angiogenesis

Another way of tumor neovascularization is intussusceptive angiogenesis, which represents a nonproliferative and noninvasive mechanism for the enlargement of a capillary plexus by intussusceptive growth, arborization, and remodeling [267] (Figure 2(C)). As this mode of vascularization is mostly independent from EC proliferation and migration, as well as BM degradation, this process is more economical and, occurring within hours or even minutes, is noticeably faster than sprouting angiogenesis [268]. It begins with the formation of transluminal pillars from the EC walls. Their subsequent expansion splits the preexisting vessel into two, thereby enhancing the vascular surface. In a subsequent process of arborization, the disorganized capillary network is remodeled into a functional tree-like structure by serial pillar formation. In a final remodeling step, the branching angles are modified, and the capillary network is pruned. The formation of new capillaries is initiated by sprouting angiogenesis that is later accompanied or followed by intussusceptive angiogenesis, which increases the EC surface [269]. Intussusceptive angiogenesis is synergistically regulated by VEGF and Ang-1, and it seems to be induced by laminar shear stress on the vessel walls, whereas oscillating shear stress favors sprouting angiogenesis [269].

#### 3.2.3. Glomeruloid Angiogenesis

In many aggressive tumors, glomeruloid angiogenesis gives rise to complex vascular structures termed glomeruloid bodies, in which several microvessels together are ensheathed by a BM of varying thickness containing sparse pericytes [270] (Figure 2(D)). The frequency of occurrence of such glomeruloid bodies is an indication for the tumor's aggressiveness and the patient's survival [271]. The formation of such glomeruloid bodies is rather a remodeling than true angiogenesis, because proliferating and migrating tumor cells can actively pull capillaries of the surrounding host vasculature and adjacent capillary branching points into the tumor node. Thereby, formed coiled vascular structures develop subsequently into glomeruloid bodies that are connected to the surrounding vasculature via numerous narrowed capillaries [256].

#### 3.2.4. Vessel Co-Option

Malignant cells can initially grow in the vicinity and along pre-existing microvessels and thus use the host vasculature for their own benefit (Figure 2(E)). This co-option of the host vasculature was originally believed to be limited to the initial phase of tumorigenesis [272]. Meanwhile, however, there is evidence that vessel co-option might persist during all stages of primary and metastatic growth of various tumors [256], for example, cutaneous melanoma, which appears to grow by co-opting the vascular plexus in its surrounding connective tissue, while there is no sign of directed vessel ingrowth [273].

Vessel co-option is regulated dependent on the tumor type and the host environment, but the key regulators are again VEGF and angiopoietins [272, 274]. Ang-1 binds to Tie-2 and thus triggers signaling cascades, assuring survival and quiescence of ECs, and thus causing tumor vessel maintenance, whereas the nonsignaling Tie-2 ligand Ang-2 acts as a negative regulator and destabilizes the capillary walls by detachment of pericytes [272, 274]. Subsequently, VEGF via its receptor VEGFR-2 promotes both survival of ECs and growth of new vessels [237, 275].

#### 3.2.5. Vascular Mimicry

Aggressive melanomas can form fluid-filled vessel-like channels without any EC lining in a nonangiogenic process termed vascular mimicry [276] (Figure 2(F)). These channels allow perfusion independent of angiogenesis, and they can arise by two types of vasculogenic mimicry, designated the tubular and the patterned matrix type [277]. These tubular vessel-like networks resemble the pattern of embryonic vascular networks, and, in their gene expression pattern, aggressive tumors that form such channels resemble endothelial, pericytes, and other precursor stem cells, suggesting that tumor cells might disguise as embryonic stem-cell-like or other cell types [256]. Vasculogenic mimicry of the patterned matrix type looks completely different and is characterized by a fluid-conducting meshwork of extravascular patterned depositions of matrix proteins such as laminins, collagens IV and VI, and heparin sulfate proteoglycans that anastomose with blood vessels [277–279]. Although it is not yet elucidated how such channels are connected to the vasculature, the latter type of vascular mimicry has been reported for many cancers, such as breast, ovarian, and prostate carcinoma, melanoma, soft tissue sarcomas, osteosarcoma, and phaeochromocytoma [277, 280]. In aggressive melanoma, the expression of tissue factor pathway-associated genes, such as tissue factor (TF), TF pathway inhibitor-1 (TFPI-1), and TFPI-2, is upregulated, suggesting an anticoagulation mechanism in the channel-forming tumor cells [281]. Fluid propelled through these channels by a pressure gradient might facilitate the supply with nutrients and oxygen, and, additionally, this fluid-conducting network could substitute for a lymphatic vascular system and drain extravasated interstitial fluid in tumors that lack lymphatic vessels, for example, uveal melanoma [279, 280].

## 4. Manipulation of Cell Matrix Interaction in Tumor Angiogenesis

Cell-matrix interactions regulate signaling pathways that are intricately interconnected with cytokine-regulated pathways, which complicates the analysis of their contribution to a particular step in angiogenesis [153]. ECM receptors can be manipulated with a wide variety of different compounds ranging from endogenous compounds, such as matrikines, over their synthetic analogues and peptides mimicking only integrin-binding sites to function-blocking antibodies and small molecules with integrin inhibitory function. Other starting points for an antiangiogenic therapy are the inhibition of signaling cascades downstream of the ECM receptors or cytokine receptors and as a new avenue the blocking of microRNAs with antisense RNAs in ECs [282, 283]. An efficient antivascular cancer therapy can target either the angiogenic signaling pathways or the vascularization mechanism [256]. A combination of conventional chemotherapy with angiosuppressive or vascular disrupting therapy is often problematic and needs careful design [256].

### 4.1. Pharmacological Intervention of Integrin-ECM Interaction

**Figure 3:**
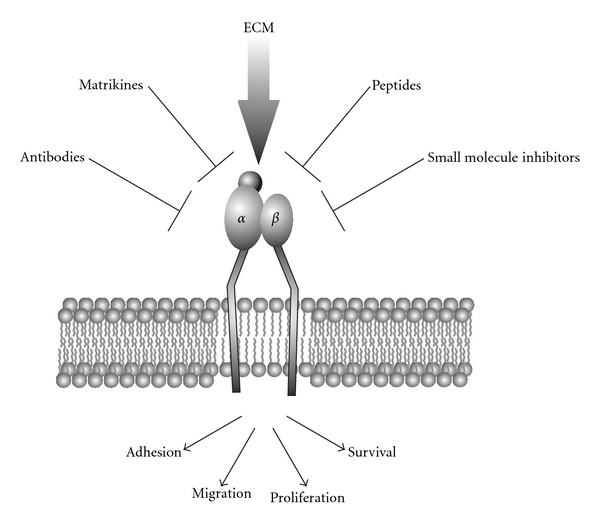
Options to manipulate integrin function. Essential cellular functions, such as adhesion, migration, proliferation, and survival, which all are regulated by integrins, can pharmacologically be manipulated with a panoply of matrikines, antibodies, peptides, and small molecule inhibitors, many of which are used as therapeutic tools in combination with conventional chemo- or radiotherapy to attack tumor cells and vasculature. Details are described in the text.

In addition to soluble growth factors, such as VEGF, there are several endogenous angiogenesis inhibitors, for example, endostatin, endorepellin, and tumstatin, which share the common feature that they all are proteolytic fragments of ECM molecules [284, 285]. In tumor angiogenesis within a primary tumor, such ECM fragments are generated by the release of MMPs, in order to degrade the BM. This results not only in labile and leaky tumor vessels but at the same time keeps metastases from growing, as these endogenous angiogenesis inhibitors are distributed via the blood stream [230]. Therefore, they are of pharmacological interest with regard to their use as angiogenesis inhibitors. Intensive efforts have been directed towards the development of integrin antagonists for the treatment of cancer and many other diseases, ranging from autoimmune diseases over inflammatory to thrombotic diseases, and their applications seem promising [11, 286]. Integrin-mediated interactions of cells with their surrounding ECM can be manipulated by antibodies, peptides, small nonpeptidic compounds, and endogenous inhibitors (Figure 3). Integrin antagonists with antiangiogenic activities have been reviewed recently with special emphasis on drugs that are in clinical trials [11].

Spurred by the success in pharmacologically targeting RGD-dependent integrins, there are also attempts to pharmacologically manipulate RGD-independent integrins, such as the collagen- and laminin-binding integrins, as reviewed recently [287]. The collagen-binding subgroup of integrins with their common A domain comprises interesting targets in the development of drugs against thrombosis, inflammatory diseases, and cancer. TSPs-1 and -2 are naturally occurring potent angiogenesis inhibitors, and their anti-angiogenic effects can be imitated by short-peptide mimetics that among other targets bind to *β1* integrins [288, 289].

An endogenous inhibitor, which blocks the interaction of integrin *α1β1* with collagen I and also binds to heparan sulfate proteoglycans, is arresten, the C-terminal fragment of the collagen IV *α1* chain [290, 291]. Endorepellin, a C-terminal fragment of perlecan specifically blocks the function of integrin *α2* *β1* [292] and interestingly also binds to endostatin, thus counteracting its antiangiogenic effect [293]. Additionally, integrin *α1β1* can be specifically inhibited with obtustatin from the snake venom of *Vipera lebetina obtusa* [294, 295]. The interaction of integrin *α2* *β1* with collagen can be specifically inhibited with the C-type lectin rhodocetin from the snake venom of *Calloselasma rhodostoma* [296, 297]. In addition, it can also be selectively antagonized by the protein angiocidin, which was first detected in lung carcinoma cells [298, 299]. The aromatic tetracyclic polyketides maggiemycin and anhydromaggiemycin from *Streptomyces*, which have been described as potential antitumor agents [300], inhibit collagen binding by blocking the A domain of the integrin subunits *α1*, *α2*, *α1* *1*, and to a lesser extent *α1* *0* while cell adhesion to fibronectin, mediated by integrins *α5β1*, *α*v*β3*, *α*v*β5*, is unaffected [301]. Recently, the sulfonamide derivative BTT-3016 has been described as a potent antithrombotic small-molecule inhibitor of integrin *α2* *β1* with only slight effect on other collagen-binding integrins and no effect on fibronectin- or vitronectin-binding integrins [302]. Another sulfonamide derivative, E7820, which does not interfere with integrin-ligand interaction, reduces integrin *α2* expression on the mRNA level [303]. Angiogenesis can be inhibited with antibodies against the *α* subunits of the integrins *α1β1* and *α2* *β1*, whereas quiescent vessels are not affected [230].

In a phase I clinical trial, endostatin, the C-terminal fragment of collagen XVIII, blocks the function of integrin *α5β1* [179, 304, 305] and also binds to heparin and with lower affinities to other heparan sulfate proteoglycans that are involved in growth factor signaling [306, 307]. Endostatin's antiangiogenic activity can also be mimicked with derived short non-RGD but arginine-rich peptides [308]. Integrin *α5β1* can also be blocked by the synthetic non-RGD peptides PHSCN, named ATN-161, [309] and cyclic CRRETAWAC [310], as well as by the peptide mimetics SJ749 [311] and JSM6427 [312], and it can be inhibited by the affinity-matured humanized chimeric monoclonal antibody M200/volociximab [313].

Angiostatin is a proteolytic fragment of plasminogen that effectively inhibits integrin *α*v*β3* [314], and its antiangiogenic effect can also be achieved by its isolated kringle-5 domain [315]. Kringle-1 to 3 show the same antiproliferative effect as the whole angiostatin, but hardly inhibit migration, whereas kringle-4 inhibits EC migration but shows only a marginal antiproliferative effect [316]. Other endogenous integrin *α*v*β3* inhibitors are the collagen XVIII fragment endostatin [304], and the C-terminal fragment of the collagen IV *α*3-chain termed tumstatin [317], which also binds to integrin *α6* *β1* [318]. Tumstatin has two binding sites for integrin *α*v*β3*. The N-terminal site mediates an antiangiogenic signal, whereas the C-terminal binding site is associated with the antitumor cellactivity [318, 319]. Canstatin, the NC1 domain of the collagen IV *α2* chain, inhibits both integrins *α*v*β3* and *α*v*β5* [320] and seems to interact with integrin *α3* *β1* too [321]. A hemopexin-like domain comprising C-terminal fragment of MMP-2, termed PEX, also antagonizes integrin *α*v*β3* by preventing its binding to MMP-2 and thus inhibiting proteolytic activity on the cell surface, especially during vessel maturation [322, 323]. Fastatin and other FAS1 domains, which are present in the four human proteins periostin, FEEL1, FEEL2, and *β*hig-h3, also function via integrin *α*v*β3* as endogenous regulators of pathogenic angiogenesis [324]. Next to these natural antagonists there is a variety of synthetic RGD—containing peptide inhibitors that mimic a motif that occurs on many ECM molecules, such as fibronectin, vitronectin, fibrinogen, osteopontin, TSP, vWF, and partially degraded collagen. Most integrins of the *α*v subfamily and the integrins *α5β1* and *α*IIb*β*3 bind to this motif. Therefore, adhesion and spreading of ECs to the ECM can be competitively inhibited by RGD peptides, whereby anchorage-dependent ECs undergo apoptosis [230]. To this group belong compounds, such as cilengitide/EMD121974 [325], S137 and S247 [326, 327], the TSP-derived peptide TP508/chrysalin [328], and several integrin *α*v*β3*- and *α*v*β5*-specific peptidomimetics, such as BCH-14661, which preferentially inhibits *α*v*β3* and BCH-15046, which blocks *α*v*β3*, *α*v*β5*, and *α*5*β1* [329], SCH221153 [330], and ST1646 [331]. Another inhibitor is the non-peptide antibiotic thiolutin, which intracellularly blocks paxillin and thus, indirectly, integrin *α*v*β3*-mediated adhesion to vitronectin [332]. Antibodies against the *β3* subunit inhibit contact of ECs to vitronectin and concomitantly VEGF-induced tyrosine phosphorylation of VEGFR-2 in cell culture studies [333]. Moreover, integrin *α*v*β3* can be effectively antagonized with the monoclonal antibody LM609/MEDI-552 and its humanized derivative abegrin/etaracizumab/vitaxin [57, 334–337]. In contrast, the humanized anti-*α*v antibody CNTO95 targets both integrins *α*v*β3* and *α*v*β5* [338]. The humanized Fab fragment 17E6/abciximab/ReoPro of the monoclonal antibody c7E3 inhibits the integrins *α*v*β3* and also *α*M*β*2/Mac-1 [339, 340], whereas the human-specific monoclonal antibody 17E6 targets all *α*v integrins [341]. Currently, humanized or chimeric integrin antibody antagonists of *α*v*β3*, *α*v*β5*, and *α5β1*, and peptide inhibitors of these integrins are in clinical trials as antiangiogenic agents [180].

## 5. Applications and Outlook

Integrins and their binding partners are of special interest as potential therapeutic targets, and several are already in clinical trials. However, the results fall short of the initial expectations, pointing out that monotherapy with a single angiogenesis inhibitor is not sufficient to counteract the numerous angiogenic factors involved in tumor progression [231]. Moreover, there are some caveats in aiming at integrins as therapeutic targets. Obviously, integrins are expressed on virtually all cells under physiological as well as pathological conditions, and it is a major challenge to target exclusively integrins on tumor or tumor-associated cells. Another problem is that low concentrations of antagonists alter the signaling of integrins and other receptors. When administered in nanomolar concentrations, the RGD-containing inhibitors cilengitide and S 36578 alter the trafficking of integrins and VEGFR2 in tumor ECs, thus stimulating angiogenesis and tumor growth [342].

Current tumor therapy aims at vessel eradication in order to disrupt the connection of the tumor to the vascular system and thus cut off the supply of nutrients and oxygen. This can be done with compounds that preferentially affect tumor endothelia rather than normal cells, that is, (i) specific angiogenesis inhibitors, (ii) tumor vessel toxins that attack inherent weaknesses in static tumor vessel endothelia and associated vascular structures, and (iii) dual-action compounds [343]. However, within the last years, a paradigm shift has taken place [344, 345]. Vessel normalization by pruning immature vessels and increasing pericytes and BM coverage of the remaining vessels comes to the fore, rather than vessel eradication, because mere antiangiogenic treatment can worsen malignancy [346]. A malformed tumor vasculature creates and aggravates a hypoxic and acidic milieu which hampers drug delivery and perfusion [347–349], and, due to its leaky endothelium, it promotes tumor cell dissemination [346]. Therefore, chemotherapeutic efficacy can be ameliorated by a concomitant vessel normalization therapy which improves delivery and efficacy of cytotoxic drugs and also sensitizes the tumor cells to radiation [345, 350].

In vessel normalization, the interaction of cells with their surrounding ECM via integrins is of special importance. However, many antiangiogenic compounds, for example, ATN-161, endostatin, and integrin inhibitors, show hormetic, that is, bell- or U-shaped, dose-response curves and thus present a challenge for clinical translation [351]. Nanomolar concentrations of RGD-mimetic *α*v*β3* and *α*v*β5* inhibitors (S 36578 and cilengitide) can paradoxically stimulate tumor growth and angiogenesis by altering the trafficking of *α*v*β3* integrin and VEGFR2. Thus, they promote the migration of ECs towards VEGF, which has important implications for the use of RGD mimetics in tumor therapy [342]. Thus, depending on tumor type, dose, and manner of application, the currently available-integrin targeting compounds can act either anti- or proangiogenic. A promising approach may be a combination therapy that blocks simultaneously angiogenic integrin *α*v*β3* and VEGFR activities [352–355].

To circumvent these problems, instead of targeting the integrins, which are in principle present on both normal and malignant cells, another strategy aims at tumor-promoting integrin ligands, such as ED-B fibronectin, tenascin-C, and tenascin W [252, 253, 255]. Invasive tumor cells partially degrade and denature their surrounding ECM, and the thereby released cryptic collagen IV epitope HU177 may also be a potential target for antiangiogenic and tumor-selective drug delivery [356].

In comparison to a systemic administration of a chemotherapeutic agent, its therapeutic index can be increased by selectively targeting integrins that are overexpressed on tumor cells [357]. Chemotherapeutic small molecules, peptides, and proteins as well as nanoparticle-carried chemotherapeutics, which are conjugated to ligands of integrins that are overexpressed on angiogenic ECs or tumor cells, can be selectively internalized after integrin binding [357]. Especially nanoparticles, such as micelles, liposomes, polymeric nanospheres, and polymersomes loaded with chemotherapeutic or radiotherapeutic drugs and equipped with multivalent integrin ligands show decreased systemic toxicity, prolonged half-life and passive retention in the tumor, improved binding affinity, and facilitated internalization, thus resulting in increased drug delivery [12, 357, 358]. A therapeutic strategy that targets several integrins and receptors by such chemo-, radio-, and possibly gene therapeutic approaches may be more effective than a monotherapy [231, 357].

Coadministration of the *α*v integrin-targeting cyclic peptide iRGD (CRGDKGPDC), or structurally closely related peptides, with anticancer drugs considerably enhances their efficacy and selectivity [359]. Upon binding to *α*v integrin-expressing tumor ECs, iRGD is proteolytically processed to CRDGK with a much weaker integrin affinity, whereas this truncated peptide shows an increased affinity to neuropilin-1 (NRP-1), thus increasing vascular and tissue permeability in a tumor-specific and NRP-1-dependent manner [359]. Interestingly, this coadministration does not require chemical conjugation of the drug with the iRGD peptide; that is, approved drugs could be used unmodified [359]. Coadministration of such a tumor-penetrating peptide with either small molecules, such as doxorubicin, antibodies, such as trastuzumab, or nanoparticles, such as Nab-paclitaxel (abraxane) or doxorubicin-loaded liposomes, resulted in equivalent or increased delivery and efficacy, and it improved their therapeutic index by lowering the effective dose [359].

Additionally, integrins can be used as biomarkers to noninvasively assess the efficacy of chemotherapeutic and radiotherapeutic drugs [12]. Integrin-targeted probes can be used to visualize tumor angiogenesis and the response to chemo- and radiotherapy by various imaging methods, such as magnetic resonance imaging (MRI), positron emission tomography (PET), and ultrasonography [360–362]. Moreover, fluorescence labeling of integrin ligands allows intraoperative fluorescence imaging, thus providing a tool to intraoperatively detect and remove metastases of submillimeter size [363].

In summary, the above data illustrate the importance of integrins and integrin-binding and signaling proteins in both physiological and pathological blood vessel formation. Thus, they may be potential targets for antiangiogenic tumor therapy. Although our knowledge concerning this matter has increased remarkably within the last years, the understanding is far from complete.

## Abbreviations

Ang: Angiopoietin
BM: Basement membrane
CRDGK, CRGDKGPDC, CRRETAWAC, PHSCN, RGD: Amino acid sequences in single letter code
ECM: Extracellular matrix
EC: Endothelial cell
FAK: Focal adhesion kinase
FGF: Fibroblast growth factor
G-CSF: Granulocyte colony-stimulating factor
HGF: Hepatocyte growth factor
HIF: Hypoxia-inducible factor
IL: Interleukin
MAPK: Mitogen-activated protein kinase
MEK: MAPK/ERK kinase
MMP: Matrix metalloproteinase
NC: Noncollagenous
NF-*κ*B: Nuclear factor *κ*-light-chain enhancer of activated B cells
NRP-1: Neuropilin-1
PDGF: Platelet-derived growth factor
PD-ECGF: Platelet-derived endothelial cell growth factor
PGE: Prostaglandin E
PI3K: Phosphatidylinositol-3 kinase
PLC: Phospholipase C
PlGF: Placenta-derived growth factor
Ras: Rat sarcoma protein
Src: Sarcoma oncogene
TGF: Transforming growth factor
Tie: Tyrosine kinase with immunoglobulin-like and EGF-like domain
TIMP: Tissue inhibitor of metalloproteinases
TNF: Tumor necrosis factor
uPA(R): Urokinase-type plasminogen activator (receptor)
VCAM: Vascular cell adhesion molecule
VEGF(R): Vascular endothelial growth factor (receptor)
VSMC: Vascular smooth muscle cell
vWF: Von Willebrand factor
TSP: Thrombospondin.

## References

[1] Kinzler K, Vogelstein B (2002). *The Genetic Basis of Human Cancer*.

[2] Bergers G, Benjamin LE (2003). Tumorigenesis and the angiogenic switch. *Nature Reviews Cancer*.

[3] Rheingold S, Neugut A, Meadows A, Kufe D, Pollock R, Weichselbaum R (2003). Secondary cancers: incidence, risk factors, and management. *Holland-Frei Cancer Medicine*.

[4] Hanahan D, Weinberg RA (2000). The hallmarks of cancer. *Cell*.

[5] Hanahan D, Weinberg RA (2011). Hallmarks of cancer: the next generation. *Cell*.

[6] Hanahan D, Folkman J (1996). Patterns and emerging mechanisms of the angiogenic switch during tumorigenesis. *Cell*.

[7] Nico B, Crivellato E, Guidolin D (2010). Intussusceptive microvascular growth in human glioma. *Clinical and Experimental Medicine*.

[8] Avraamides CJ, Garmy-Susini B, Varner JA (2008). Integrins in angiogenesis and lymphangiogenesis. *Nature Reviews Cancer*.

[9] Rathinam R, Alahari SK (2010). Important role of integrins in the cancer biology. *Cancer and Metastasis Reviews*.

[10] Alghisi G, Rüegg C (2006). Vascular integrins in tumor angiogenesis: mediators and therapeutic targets. *Endothelium*.

[11] Rüegg C, Alghisi GC (2010). Vascular integrins: therapeutic and imaging targets of tumor angiogenesis. *Recent Results in Cancer Research*.

[12] Desgrosellier JS, Cheresh DA (2010). Integrins in cancer: biological implications and therapeutic opportunities. *Nature Reviews Cancer*.

[13] Kim S-H, Turnbull J, Guimond S (2011). Extracellular matrix and cell signalling: the dynamic cooperation of integrin, proteoglycan and growth factor receptor. *Journal of Endocrinology*.

[14] Gartner L, Hiat J (1994). *Color Atlas of Histology*.

[15] Eble JA, Niland S (2009). The extracellular matrix of blood vessels. *Current Pharmaceutical Design*.

[16] LeBleu VS, MacDonald B, Kalluri R (2007). Structure and function of basement membranes. *Experimental Biology and Medicine*.

[17] Iozzo RV, Zoeller JJ, Nyström A (2009). Basement membrane proteoglycans: modulators Par excellence of cancer growth and angiogenesis. *Molecules and Cells*.

[18] Yurchenco PD (2011). Basement membranes: cell scaffoldings and signaling platforms. *Cold Spring Harbor Perspectives in Biology*.

[19] Astrof S, Hynes RO (2009). Fibronectins in vascular morphogenesis. *Angiogenesis*.

[20] Katsuda S, Kaji T (2003). Atherosclerosis and extracellular matrix. *Journal of Atherosclerosis and Thrombosis*.

[21] Plenz GAM, Deng MC, Robenek H, Völker W (2003). Vascular collagens: spotlight on the role of type VIII collagen in atherogenesis. *Atherosclerosis*.

[22] Kühn K (1995). Basement membrane (type IV) collagen. *Matrix Biology*.

[23] Pöschl E, Schlötzer-Schrehardt U, Brachvogel B, Saito K, Ninomiya Y, Mayer U (2004). Collagen IV is essential for basement membrane stability but dispensable for initiation of its assembly during early development. *Development*.

[24] Mithieux SM, Weiss AS (2005). Elastin. *Advances in Protein Chemistry*.

[25] Kielty CM (2006). Elastic fibres in health and disease. *Expert Reviews in Molecular Medicine*.

[26] Patel A, Fine B, Sandig M, Mequanint K (2006). Elastin biosynthesis: the missing link in tissue-engineered blood vessels. *Cardiovascular Research*.

[27] Colombatti A, Doliana R, Bot S (2000). The EMILIN protein family. *Matrix Biology*.

[28] Brooke BS, Karnik SK, Li DY (2003). Extracellular matrix in vascular morphogenesis and disease: structure versus signal. *Trends in Cell Biology*.

[29] Timpl R, Sasaki T, Kostka G, Chu ML (2003). Fibulins: a versatile family of extracellular matrix proteins. *Nature Reviews Molecular Cell Biology*.

[30] Nakamura T, Lozano PR, Ikeda Y (2002). Fibulin-5/DANCE is essential for elastogenesis in vivo. *Nature*.

[31] Hirai M, Ohbayashi T, Horiguchi M (2007). Fibulin-5/DANCE has an elastogenic organizer activity that is abrogated by proteolytic cleavage in vivo. *Journal of Cell Biology*.

[32] Giltay R, Timpl R, Kostka G (1999). Sequence, recombinant expression and tissue localization of two novel extracellular matrix proteins, fibulin-3 and fibulin-4. *Matrix Biology*.

[33] Dingemans KP, Teeling P, Lagendijk JH, Becker AE (2000). Extracellular matrix of the human aortic media: an ultrastructural histochemical and immunohistochemical study of the adult aortic media. *Anatomical Record*.

[34] Marneros AG, Olsen BR (2005). Physiological role of collagen XVIII and endostatin. *FASEB Journal*.

[35] Grässel S, Unsöld C, Schäcke H, Bruckner-Tuderman L, Bruckner P (1999). Collagen XVI is expressed by human dermal fibroblasts and keratinocytes and is associated with the microfibrillar apparatus in the upper papillary dermis. *Matrix Biology*.

[36] Kassner A, Hansen U, Miosge N (2003). Discrete integration of collagen XVI into tissue-specific collagen fibrils or beaded microfibrils. *Matrix Biology*.

[37] Moiseeva EP (2001). Adhesion receptors of vascular smooth muscle cells and their functions. *Cardiovascular Research*.

[38] Eble JA, Kassner A, Niland S, Mörgelin M, Grifka J, Grässel S (2006). Collagen XVI harbors an integrin *α*1*β*1 recognition site in its C-terminal domains. *Journal of Biological Chemistry*.

[39] Thyberg J, Blomgren K, Roy J, Tran PK, Hedin U (1997). Phenotypic modulation of smooth muscle cells after arterial injury is associated with changes in the distribution of laminin and fibronectin. *Journal of Histochemistry and Cytochemistry*.

[40] Hall AP (2006). Review of the pericyte during angiogenesis and its role in cancer and diabetic retinopathy. *Toxicologic Pathology*.

[41] Adams RH, Alitalo K (2007). Molecular regulation of angiogenesis and lymphangiogenesis. *Nature Reviews Molecular Cell Biology*.

[42] Gerhardt H, Semb H (2008). Pericytes: gatekeepers in tumour cell metastasis?. *Journal of Molecular Medicine*.

[43] Isogai Z, Aspberg A, Keene DR, Ono RN, Reinhardt DP, Sakai LY (2002). Versican interacts with fibrillin-1 and links extracellular microfibrils to other connective tissue networks. *Journal of Biological Chemistry*.

[44] Aspberg A, Adam S, Kostka G, Timpl R, Heinegård D (1999). Fibulin-1 is a ligand for the C-type lectin domains of aggrecan and versican. *Journal of Biological Chemistry*.

[45] Olin AI, Mörgelin M, Sasaki T, Timpl R, Heinegård D, Aspberg A (2001). The proteoglycans aggrecan and versican form networks with fibulin-2 through their lectin domain binding. *Journal of Biological Chemistry*.

[46] Heino J, Käpylä J (2009). Cellular receptors of extracellular matrix molecules. *Current Pharmaceutical Design*.

[47] Contois L, Akalu A, Brooks PC (2009). Integrins as “functional hubs” in the regulation of pathological angiogenesis. *Seminars in Cancer Biology*.

[48] Zaidel-Bar R, Itzkovitz S, Ma’ayan A, Iyengar R, Geiger B (2007). Functional atlas of the integrin adhesome. *Nature Cell Biology*.

[49] Silva R, D’Amico G, Hodivala-Dilke KM, Reynolds LE (2008). Integrins: the keys to unlocking angiogenesis. *Arteriosclerosis, Thrombosis, and Vascular Biology*.

[50] Miettinen M, Castello R, Wayner E, Schwarting R (1993). Distribution of VLA integrins in solid tumors: emergence of tumor-type- related expression patterns in carcinomas and sarcomas. *American Journal of Pathology*.

[51] Senger DR, Claffey KP, Benes JE, Perruzzi CA, Sergiou AP, Detmar M (1997). Angiogenesis promoted by vascular endothelial growth factor: regulation through *α*1*β*1 and *α*2*β*1 integrins. *Proceedings of the National Academy of Sciences of the United States of America*.

[52] Senger DR, Perruzzi CA, Streit M, Koteliansky VE, De Fougerolles AR, Detmar M (2002). The *α*1*β*1 and *α*2*β*1 integrins provide critical support for vascular endothelial growth factor signaling, endothelial cell migration, and tumor angiogenesis. *American Journal of Pathology*.

[53] Vuoriluoto K, Högnäs G, Meller P, Lehti K, Ivaska J (2011). Syndecan-1 and -4 differentially regulate oncogenic K-ras dependent cell invasion into collagen through *α*2*β*1 integrin and MT1-MMP. *Matrix Biology*.

[54] Hynes RO (2002). Integrins: bidirectional, allosteric signaling machines. *Cell*.

[55] Yang JT, Rayburn H, Hynes RO (1995). Cell adhesion events mediated by *α*4 integrins are essential in placental and cardiac development. *Development*.

[56] Morgan MR, Humphries MJ, Bass MD (2007). Synergistic control of cell adhesion by integrins and syndecans. *Nature Reviews Molecular Cell Biology*.

[57] Brooks PC, Clark RAF, Cheresh DA (1994). Requirement of vascular integrin *α*(v)*β*3 for angiogenesis. *Science*.

[58] Hynes RO (2007). Cell-matrix adhesion in vascular development. *Journal of Thrombosis and Haemostasis*.

[59] Landen CN, Kim TJ, Lin YG (2008). Tumor-selective response to antibody-mediated targeting of *α*v*β*3 integrin in ovarian cancer. *Neoplasia*.

[60] Davis GE (1992). Affinity of integrins for damaged extracellular matrix: *α*(v)*β*3 binds to denatured collagen type I through RGD sites. *Biochemical and Biophysical Research Communications*.

[61] Nieswandt B, Hafner M, Echtenacher B, Männel DN (1999). Lysis of tumor cells by natural killer cells in mice is impeded by platelets. *Cancer Research*.

[62] Brooks PC, Stromblad S, Klemke R, Visscher D, Sarkar FH, Cheresh DA (1995). Antiintegrin *α*v*β*3 blocks human breast cancer growth and angiogenesis in human skin. *Journal of Clinical Investigation*.

[63] Mahabeleshwar GH, Feng W, Phillips DR, Byzova TV (2006). Integrin signaling is critical for pathological angiogenesis. *Journal of Experimental Medicine*.

[64] Huang X, Griffiths M, Wu J, Farese RV, Sheppard D (2000). Normal development, wound healing, and adenovirus susceptibility in *β*5- deficient mice. *Molecular and Cellular Biology*.

[65] Bader BL, Rayburn H, Crowley D, Hynes RO (1998). Extensive vasculogenesis, angiogenesis, and organogenesis precede lethality in mice lacking all *α*v integrins. *Cell*.

[66] McCarty JH, Monahan-Earley RA, Brown LF (2002). Defective associations between blood vessels and brain parenchyma lead to cerebral hemorrhage in mice lacking *α*v integrins. *Molecular and Cellular Biology*.

[67] McCarty JH, Lacy-Hulbert A, Charest A (2005). Selective ablation of *α*v integrins in the central nervous system leads to cerebral hemorrhage, seizures, axonal degeneration and premature death. *Development*.

[68] Reynolds LE, Wyder L, Lively JC (2002). Enhanced pathological angiogenesis in mice lacking *β*3 integrin or *β*3 and *β*5 integrins. *Nature Medicine*.

[69] Díaz-González F, Forsyth J, Steiner B, Ginsberg MH (1996). Trans-dominant inhibition of integrin function. *Molecular Biology of the Cell*.

[70] Hodivala-Dilke KM, DiPersio CM, Kreidberg JA, Hynes RO (1998). Novel roles for *α*3*β*1 integrin as a regulator of cytoskeletal assembly and as a trans-dominant inhibitor of integrin receptor function in mouse keratinocytes. *Journal of Cell Biology*.

[71] Stupack DG, Puente XS, Boutsaboualoy S, Storgard CM, Cheresh DA (2001). Apoptosis of adherent cells by recruitment of caspase-8 to unligated integrins. *Journal of Cell Biology*.

[72] Zhu J, Motejlek K, Wang D, Zang K, Schmidt A, Reichardt LF (2002). *β*8 Integrins are required for vascular morphogenesis in mouse embryos. *Development*.

[73] Venstrom K, Reichardt L (1995). Beta 8 integrins mediate interactions of chick sensory neurons with laminin-1, collagen IV, and fibronectin. *Molecular Biology of the Cell*.

[74] Milner R, Relvas JB, Fawcett J, Ffrench-Constant C (2001). Developmental regulation of *α*v integrins produces functional changes in astrocyte behavior. *Molecular and Cellular Neuroscience*.

[75] Mu D, Cambier S, Fjellbirkeland L (2002). The integrin *ανβ*8 mediates epithelial homeostasis through MT1-MMP-dependent activation of TGF-*β*1. *Journal of Cell Biology*.

[76] Kern A, Eble J, Golbik R, Kuhn K (1993). Interaction of type IV collagen with the isolated integrins *α*1*β*1 and *α*2*β*1. *European Journal of Biochemistry*.

[77] Tulla M, Pentikäinen OT, Viitasalo T (2001). Selective binding of collagen subtypes by integrin *α*1I, *α*2I, and *α*10I domains. *Journal of Biological Chemistry*.

[78] Zutter MM, Santoro SA (1990). Widespread histologic distribution of the *α*2*β*1 integrin cell-surface collagen receptor. *American Journal of Pathology*.

[79] Bengtsson T, Aszodi A, Nicolae C, Hunziker EB, Lundgren-Åkerlund E, Fässler R (2005). Loss of *α*10*β*1 integrin expression leads to moderate dysfunction of growth plate chondrocytes. *Journal of Cell Science*.

[80] Popova SN, Barczyk M, Tiger CF (2007). *α*11*β*1 integrin-dependent regulation of periodontal ligament function in the erupting mouse incisor. *Molecular and Cellular Biology*.

[81] Riikonen T, Westermarck J, Koivisto L, Broberg A, Kahari VM, Heino J (1995). Integrin *α*2*β*1 is a positive regulator of collagenase (MMP-1) and collagen *α*1(I) gene expression. *Journal of Biological Chemistry*.

[82] Langholz O, Röckel D, Mauch C (1995). Collagen and collagenase gene expression in three-dimensional collagen lattices are differentially regulated by *α*1*β*1 and *α*2*β*1 integrins. *Journal of Cell Biology*.

[83] Gardner H, Broberg A, Pozzi A, Laato M, Heino J (1999). Absence of integrin *α*1*β*1 in the mouse causes loss of feedback regulation of collagen synthesis in normal and wounded dermis. *Journal of Cell Science*.

[84] Alves F, Vogel W, Mossie K, Millauer B, Hofler H, Ullrich A (1995). Distinct structural characteristics of discoidin I subfamily receptor tyrosine kinases and complementary expression in human cancer. *Oncogene*.

[85] Auger JM, Kuijpers MJE, Senis YA, Watson SP, Heemskerk JWM (2005). Adhesion of human and mouse platelets to collagen under shear: a unifying model. *FASEB Journal*.

[86] Meyaard L (2008). The inhibitory collagen receptor LAIR-1 (CD305). *Journal of Leukocyte Biology*.

[87] Curino AC, Engelholm LH, Yamada SS (2005). Intracellular collagen degradation mediated by uPARAP/Endo180 is a major pathway of extracellular matrix turnover during malignancy. *Journal of Cell Biology*.

[88] Kreidberg JA, Donovan MJ, Goldstein SL (1996). Alpha 3 beta 1 integrin has a crucial role in kidney and lung organogenesis. *Development*.

[89] DiPersio CM, Hodivala-Dilke KM, Jaenisch R, Kreidberg JA, Hynes RO (1997). *α*3*β*1 integrin is required for normal development of the epidermal basement membrane. *Journal of Cell Biology*.

[90] Mayer U, Saher G, Fässler R (1997). Absence of integrin *α*7 causes a novel form of muscular dystrophy. *Nature Genetics*.

[91] Stepp MA, Spurr-Michaud S, Tisdale A, Elwell J, Gipson IK (1990). *α*6*β*4 integrin heterodimer is a component of hemidesmosomes. *Proceedings of the National Academy of Sciences of the United States of America*.

[92] Ibraghimov-Beskrovnaya O, Ervasti JM, Leveille CJ, Slaughter CA, Sernett SW, Campbell KP (1992). Primary structure of dystrophin-associated glycoproteins linking dystrophin to the extracellular matrix. *Nature*.

[93] Haenggi T, Fritschy JM (2006). Role of dystrophin and utrophin for assembly and function of the dystrophin glycoprotein complex in non-muscle tissue. *Cellular and Molecular Life Sciences*.

[94] Nelson J, McFerran NV, Pivato G (2008). The 67 kDa laminin receptor: structure, function and role in disease. *Bioscience reports*.

[95] Fontanini G, Vignati S, Chiné S (1997). 67-kilodalton laminin receptor expression correlates with worse prognostic indicators in non-small cell lung carcinomas. *Clinical Cancer Research*.

[96] Waltregny D, De Leval L, Ménard S, De Leval J, Castronovo V (1997). Independent prognostic value of the 67-kd laminin receptor in human prostate cancer. *Journal of the National Cancer Institute*.

[97] Ardini E, Tagliabue E, Magnifico A (1997). Co-regulation and physical association of the 67-kDa monomeric laminin receptor and the *α*6*β*4 integrin. *Journal of Biological Chemistry*.

[98] Ogawa T, Tsubota Y, Hashimoto J, Kariya Y, Miyazaki K (2007). The short arm of laminin *γ*2 chain of laminin-5 (laminin-332) binds syndecan-1 and regulates cellular adhesion and migration by suppressing phosphorylation of integrin *β*4 chain. *Molecular Biology of the Cell*.

[99] Da Silva RG, Tavora B, Robinson SD (2010). Endothelial *α*3*β*1-integrin represses pathological angiogenesis and sustains endothelial-VEGF. *American Journal of Pathology*.

[100] Van der Neut R, Krimpenfort P, Calafat J, Niessen CM, Sonnenberg A (1996). Epithelial detachment due to absence of hemidesmosomes in integrin *β* null mice. *Nature Genetics*.

[101] Nikolopoulos SN, Blaikie P, Yoshioka T, Guo W, Giancotti FG (2004). Integrin *β*4 signaling promotes tumor angiogenesis. *Cancer Cell*.

[102] Hiran TS, Mazurkiewicz JE, Kreienberg P, Rice FL, LaFlamme SE (2003). Endothelial expression of the *α*6*β*4 integrin is negatively regulated during angiogenesis. *Journal of Cell Science*.

[103] Frisch SM, Francis H (1994). Disruption of epithelial cell-matrix interactions induces apoptosis. *Journal of Cell Biology*.

[104] Wickström SA, Radovanac K, Fässler R (2011). Genetic analyses of integrin signaling. *Cold Spring Harbor Perspectives in Biology*.

[105] Giancotti FG, Ruoslahti E (1999). Integrin signaling. *Science*.

[106] Arnaout MA, Mahalingam B, Xiong JP (2005). Integrin structure, allostery, and bidirectional signaling. *Annual Review of Cell and Developmental Biology*.

[107] Methe H, Hess S, Edelman ER (2007). Endothelial immunogenicity—a matter of matrix microarchitecture. *Thrombosis and Haemostasis*.

[108] Wallez Y, Huber P (2008). Endothelial adherens and tight junctions in vascular homeostasis, inflammation and angiogenesis. *Biochimica et Biophysica Acta*.

[109] Orpana A, Ranta V, Mikkola T, Viinikka L, Ylikorkala O (1997). Inducible nitric oxide and prostacyclin productions are differently controlled by extracellular matrix and cell density in human vascular endothelial cells. *Journal of Cellular Biochemistry*.

[110] Schwartz MA, DeSimone DW (2008). Cell adhesion receptors in mechanotransduction. *Current Opinion in Cell Biology*.

[111] Ivaska J, Heino J (2010). Interplay between cell adhesion and growth factor receptors: from the plasma membrane to the endosomes. *Cell and Tissue Research*.

[112] Hutchings H, Ortega N, Plouët J (2003). Extracellular matrix-bound vascular endothelial growth factor promotes endothelial cell adhesion, migration, and survival through integrin ligation. *The FASEB Journal*.

[113] Vlahakis NE, Young BA, Atakilit A (2007). Integrin *α*9*β*1 directly binds to vascular endothelial growth factor (VEGF)-A and contributes to VEGF-A-induced angiogenesis. *Journal of Biological Chemistry*.

[114] Vlahakis NE, Young BA, Atakilit A, Sheppard D (2005). The lymphangiogenic vascular endothelial growth factors VEGF-C and -D are ligands for the integrin *α*9*β*1. *Journal of Biological Chemistry*.

[115] Carlson TR, Feng Y, Maisonpierre PC, Mrksich M, Morla AO (2001). Direct cell adhesion to the angiopoietins mediated by integrins. *Journal of Biological Chemistry*.

[116] Leu SJ, Lam SCT, Lau LF (2002). Pro-angiogenic activities of CYR61 (CCN1) mediated through integrins *α*v*β*3 and *α*6*β*1 in human umbilical vein endothelial cells. *Journal of Biological Chemistry*.

[117] Leu SJ, Liu Y, Chen N, Chen CC, Lam SCT, Lau LF (2003). Identification of a novel integrin *α*6*β*1 binding site in the angiogenic inducer CCN1 (CYR61). *Journal of Biological Chemistry*.

[118] Mori S, Wu CY, Yamaji S (2008). Direct binding of integrin *α*v*β*3 to FGF1 plays a role in FGF1 signaling. *Journal of Biological Chemistry*.

[119] Suzuki K, Okuno T, Yamamoto M (2007). Semaphorin 7A initiates T-cell-mediated inflammatory responses through *α*1*β*1 integrin. *Nature*.

[120] Chao JT, Martinez-Lemus LA, Kaufman SJ, Meininger GA, Ramos KS, Wilson E (2006). Modulation of *α*7-integrin-mediated adhesion and expression by platelet-derived growth factor in vascular smooth muscle cells. *American Journal of Physiology*.

[121] Flintoff-Dye NL, Welser J, Rooney J (2005). Role for the *α*7*β*1 integrin in vascular development and integrity. *Developmental Dynamics*.

[122] Taooka Y, Chen J, Yednock T, Sheppard D (1999). The integrin *α*9*β*1 mediates adhesion to activated endothelial cells and transendothelial neutrophil migration through interaction with vascular cell adhesion molecule-1. *Journal of Cell Biology*.

[123] Staniszewska I, Zaveri S, Valle LD (2007). Interaction of *α*9*β*1 integrin with thrombospondin-1 promotes angiogenesis. *Circulation Research*.

[124] Bouvard D, Brakebusch C, Gustafsson E (2001). Functional consequences of integrin gene mutations in mice. *Circulation Research*.

[125] Fässler R, Meyer M (1995). Consequences of lack of *β*1 integrin gene expression in mice. *Genes and Development*.

[126] Stephens LE, Sutherland AE, Klimanskaya IV (1995). Deletion of *β*1 integrins in mice results in inner cell mass failure and peri-implantation lethality. *Genes and Development*.

[127] Carlson TR, Hu H, Braren R, Kim YH, Wang RA (2008). Cell-autonomous requirement for *β*1 integrin in endothelial cell adhesion, migration and survival during angiogenesis in mice. *Development*.

[128] Lei L, Liu D, Huang Y (2008). Endothelial expression of *β*1 integrin is required for embryonic vascular patterning and postnatal vascular remodeling. *Molecular and Cellular Biology*.

[129] Tanjore H, Zeisberg EM, Gerami-Naini B, Kalluri R (2008). *β*1 integrin expression on endothelial cells is required for angiogenesis but not for vasculogenesis. *Developmental Dynamics*.

[130] Zovein AC, Luque A, Turlo KA (2010). *β*1 integrin establishes endothelial cell polarity and arteriolar lumen formation via a Par3-dependent mechanism. *Developmental Cell*.

[131] Pozzi A, Moberg PE, Miles LA, Wagner S, Soloway P, Gardner HA (2000). Elevated matrix metalloprotease and angiostatin levels in integrin *α*1 knockout mice cause reduced tumor vascularization. *Proceedings of the National Academy of Sciences of the United States of America*.

[132] Zhang Z, Ramirez NE, Yankeelov TE (2008). *α*2*β*1 integrin expression in the tumor microenvironment enhances tumor angiogenesis in a tumor cell-specific manner. *Blood*.

[133] Yang JT, Rayburn H, Hynes RO (1993). Embryonic mesodermal defects in *α*5 integrin-deficient mice. *Development*.

[134] Francis SE, Goh KL, Hodivala-Dilke K (2002). Central roles of *α*5*β*1 integrin and fibronectin in vascular development in mouse embryos and embryoid bodies. *Arteriosclerosis, Thrombosis, and Vascular Biology*.

[135] Parsons-Wingerter P, Kasman IM, Norberg S (2005). Uniform overexpression and rapid accessibility of *α*5*β*1 integrin on blood vessels in tumors. *American Journal of Pathology*.

[136] Georges-Labouesse E, Messaddeq N, Yehia G, Cadalbert L, Dierich A, Le Meur M (1996). Absence of integrin *α*6 leads to epidermolysis bullosa and neonatal death in mice. *Nature Genetics*.

[137] Welser JV, Lange ND, Flintoff-Dye N, Burkin HR, Burkin DJ (2007). Placental Defects in *α*7 Integrin Null Mice. *Placenta*.

[138] Fässler R, Georges-Labouesse E, Hirsch E (1996). Genetic analyses of integrin function in mice. *Current Opinion in Cell Biology*.

[139] Huang XZ, Wu JF, Ferrando R (2000). Fatal bilateral chylothorax in mice lacking the integrin *α*9*β*1. *Molecular and Cellular Biology*.

[140] Hodivala-Dilke KM, McHugh KP, Tsakiris DA (1999). *β*3-integrin-deficient mice are a model for Glanzmann thrombasthenia showing placental defects and reduced survival. *Journal of Clinical Investigation*.

[141] Takada Y, Ye X, Simon S (2007). The integrins. *Genome Biology*.

[142] Ginsberg MH, Partridge A, Shattil SJ (2005). Integrin regulation. *Current Opinion in Cell Biology*.

[143] Arnaout MA, Goodman SL, Xiong JP (2007). Structure and mechanics of integrin-based cell adhesion. *Current Opinion in Cell Biology*.

[144] Luo BH, Carman CV, Springer TA (2007). Structural basis of integrin regulation and signaling. *Annual Review of Immunology*.

[145] Takagi J, Strokovich K, Springer TA, Walz T (2003). Structure of integrin *α*5*β*1 in complex with fibronectin. *EMBO Journal*.

[146] Xiong JP, Stehle T, Diefenbach B (2001). Crystal structure of the extracellular segment of integrin *α*V*β*3. *Science*.

[147] Xiong JP, Stehle T, Goodman SL, Arnaout MA (2003). New insights into the structural basis of integrin activation. *Blood*.

[148] Xiong J-P, Stehle T, Zhang R (2002). Crystal structure of the extracellular segment of integrin *α*V*β*3 in complex with an Arg-Gly-Asp ligand. *Science*.

[149] Humphries MJ, Symonds EJH, Mould AP (2003). Mapping functional residues onto integrin crystal structures. *Current Opinion in Structural Biology*.

[150] Mould AP, Koper EJ, Byron A, Zahn G, Humphries MJ (2009). Mapping the ligand-binding pocket of integrin *α*5*β*1 using a gain-of-function approach. *Biochemical Journal*.

[151] Calderwood DA (2004). Integrin activation. *Journal of Cell Science*.

[152] Shattil SJ, Kim C, Ginsberg MH (2010). The final steps of integrin activation: the end game. *Nature Reviews Molecular Cell Biology*.

[153] Ramjaun AR, Hodivala-Dilke K (2009). The role of cell adhesion pathways in angiogenesis. *International Journal of Biochemistry and Cell Biology*.

[154] Wegener KL, Partridge AW, Han J (2007). Structural Basis of Integrin Activation by Talin. *Cell*.

[155] Moser M, Nieswandt B, Ussar S, Pozgajova M, Fässler R (2008). Kindlin-3 is essential for integrin activation and platelet aggregation. *Nature Medicine*.

[156] Zhu J, Carman CV, Kim M, Shimaoka M, Springer TA, Luo BH (2007). Requirement of *α* and *β* subunit transmembrane helix separation for integrin outside-in signaling. *Blood*.

[157] Xiao T, Takagi J, Coller BS, Wang JH, Springer TA (2004). Structural basis for allostery in integrins and binding to fibrinogen-mimetic therapeutics. *Nature*.

[158] Nishida N, Xie C, Shimaoka M, Cheng Y, Walz T, Springer TA (2006). Activation of leukocyte *β*2 integrins by conversion from bent to extended conformations. *Immunity*.

[159] Legate KR, Montañez E, Kudlacek O, Fässler R (2006). ILK, PINCH and parvin: the tIPP of integrin signalling. *Nature Reviews Molecular Cell Biology*.

[160] Zöller M (2009). Tetraspanins: push and pull in suppressing and promoting metastasis. *Nature Reviews Cancer*.

[161] Park JH, Ryu JM, Han HJ (2011). Involvement of caveolin-1 in fibronectin-induced mouse embryonic stem cell proliferation: role of FAK, RhoA, PI3K/Akt, and ERK 1/2 pathways. *Journal of Cellular Physiology*.

[162] Lee SH, Lee YJ, Park SW, Kim HS, Han HJ (2011). Caveolin-1 and integrin *β*1 regulate embryonic stem cell proliferation via p38 MAPK and FAK in high glucose. *Journal of Cellular Physiology*.

[163] Byron A, Morgan MR, Humphries MJ (2010). Adhesion signalling complexes. *Current Biology*.

[164] Del Pozo MA, Alderson NB, Kiosses WB, Chiang HH, Anderson RGW, Schwartz MA (2004). Integrins regulate rac targeting by internalization of membrane domains. *Science*.

[165] Salanueva IJ, Cerezo A, Guadamillas MC, Del Pozo MA (2007). Integrin regulation of caveolin function: caveolae review series. *Journal of Cellular and Molecular Medicine*.

[166] Bethani I, Skånland SS, Dikic I, Acker-Palmer A (2010). Spatial organization of transmembrane receptor signalling. *EMBO Journal*.

[167] Tilghman RW, Parsons JT (2008). Focal adhesion kinase as a regulator of cell tension in the progression of cancer. *Seminars in Cancer Biology*.

[168] Brown MC, Cary LA, Jamieson JS, Cooper JA, Turner CE (2005). Src and FAK kinases cooperate to phosphorylate paxillin kinase linker, stimulate its focal adhesion localization, and regulate cell spreading and protrusiveness. *Molecular Biology of the Cell*.

[169] Arias-Salgado EG, Lizano S, Sarkar S, Brugge JS, Ginsberg MH, Shattil SJ (2003). Src kinase activation by direct interaction with the integrin *β* cytoplasmic domain. *Proceedings of the National Academy of Sciences of the United States of America*.

[170] Hanks SK, Calalb MB, Harper MC, Patel SK (1992). Focal adhesion protein-tyrosine kinase phosphorylated in response to cell attachment to fibronectin. *Proceedings of the National Academy of Sciences of the United States of America*.

[171] Papapetropoulos A, Fulton D, Mahboubi K (2000). Angiopoietin-1 inhibits endothelial cell apoptosis via the Akt/survivin pathway. *Journal of Biological Chemistry*.

[172] Kim SH, Kim SH (2008). Antagonistic effect of EGF on FAK phosphorylation/dephosphorylation in a cell. *Cell Biochemistry and Function*.

[173] Slack-Davis JK, Eblen ST, Zecevic M (2003). PAK1 phosphorylation of MEK1 regulates fibronectin-stimulated MAPK activation. *Journal of Cell Biology*.

[174] Edin ML, Juliano RL (2005). Raf-1 serine 338 phosphorylation plays a key role in adhesion-dependent activation of extracellular signal-regulated kinase by epidermal growth factor. *Molecular and Cellular Biology*.

[175] Shen TL, Park AYJ, Alcaraz A (2005). Conditional knockout of focal adhesion kinase in endothelial cells reveals its role in angiogenesis and vascular development in late embryogenesis. *Journal of Cell Biology*.

[176] Courter DL, Lomas L, Scatena M, Giachelli CM (2005). Src kinase activity is required for integrin *α*v*β* 3-mediated activation of nuclear factor-*κ*B. *Journal of Biological Chemistry*.

[177] Zaric J, Rüegg C (2005). Integrin-mediated adhesion and soluble ligand binding stabilize COX-2 protein levels in endothelial cells by inducing expression and preventing degradation. *Journal of Biological Chemistry*.

[178] Eliceiri BP, Puente XS, Hood JD (2002). Src-mediated coupling of focal adhesion kinase to integrin *α*v*β*5 in vascular endothelial growth factor signaling. *Journal of Cell Biology*.

[179] Wickström SA, Alitalo K, Keski-Oja J (2002). Endostatin associates with integrin *α*5*β*1 and caveolin-1, and activates Src via a tyrosyl phosphatase-dependent pathway in human endothelial cells. *Cancer Research*.

[180] Aiyer A, Varner J, Teicher BA, Ellis LM (2008). The role of integrins in tumor angiogenesis. *Cancer Drug Discovery Development—Antiangiogenic Agents in Cancer Therapy*.

[181] Chandra Kumar C (1998). Signaling by integrin receptors. *Oncogene*.

[182] Nagashima KI, Endo A, Ogita H (2002). Adaptor protein Crk is required for ephrin-B1-induced membrane ruffling and focal complex assembly of human aortic endothelial cells. *Molecular Biology of the Cell*.

[183] Paulhe F, Racaud-Sultan C, Ragab A (2001). Differential regulation of phosphoinositide metabolism by *α* v*β*3 and *α*v*β*5 integrins upon smooth muscle cell migration. *Journal of Biological Chemistry*.

[184] Carloni V, Romanelli RG, Pinzani M, Laffi G, Gentilini P (1997). Focal adhesion kinase and phospholipase C*γ* involvement in adhesion and migration of human hepatic stellate cells. *Gastroenterology*.

[185] Zhang X, Chattopadhyay A, Ji QS (1999). Focal adhesion kinase promotes phospholipase C-*γ*1 activity. *Proceedings of the National Academy of Sciences of the United States of America*.

[186] Bi L, Okabe I, Bernard DJ, Wynshaw-Boris A, Nussbaum RL (1999). Proliferative defect and embryonic lethality in mice homozygous for a deletion in the p110*α* subunit of phosphoinositide 3-kinase. *Journal of Biological Chemistry*.

[187] Lelievre E, Bourbon PM, Duan LJ, Nussbaum RL, Fong GH (2005). Deficiency in the p110*α* subunit of PI3K results in diminished Tie2 expression and Tie2-/–like vascular defects in mice. *Blood*.

[188] Graupera M, Guillermet-Guibert J, Foukas LC (2008). Angiogenesis selectively requires the p110*α* isoform of PI3K to control endothelial cell migration. *Nature*.

[189] Sudhakar A, Sugimoto H, Yang C, Lively J, Zeisberg M, Kalluri R (2003). Human tumstatin and human endostatin exhibit distinct antiangiogenic activities mediated by *α*v*β* and *α*5*β*1 integrins. *Proceedings of the National Academy of Sciences of the United States of America*.

[190] Gupta S, Ramjaun AR, Haiko P (2007). Binding of ras to phosphoinositide 3-kinase p110*α* is required for ras-driven tumorigenesis in mice. *Cell*.

[191] Roberts MS, Woods AJ, Shaw PE, Norman JC (2003). ERK1 associates with *α*v*β*3 integrin and regulates cell spreading on vitronectin. *Journal of Biological Chemistry*.

[192] Short SM, Talbott GA, Juliano RL (1998). Integrin-mediated signaling events in human endothelial cells. *Molecular Biology of the Cell*.

[193] Hüser M, Luckett J, Chiloeches A (2001). MEK kinase activity is not necessary for Raf-1 function. *EMBO Journal*.

[194] Giroux S, Tremblay M, Bernard D (1999). Embryonic death of Mek1-deficient mice reveals a role for this kinase in angiogenesis in the labyrinthine region of the placenta. *Current Biology*.

[195] Hood JD, Frausto R, Kiosses WB, Schwartz MA, Cheresh DA (2003). Differential *α*v integrin-mediated Ras-ERK signaling during two pathways of angiogenesis. *Journal of Cell Biology*.

[196] Wary KK, Mainiero F, Isakoff SJ, Marcantonio EE, Giancotti FG (1996). The adaptor protein Shc couples a class of integrins to the control of cell cycle progression. *Cell*.

[197] Pozzi A, Wary KK, Giancotti FG, Gardner HA (1998). Integrin *α*1*β*1 mediates a unique collagen-dependent proliferation pathway in vivo. *Journal of Cell Biology*.

[198] Fournier AK, Campbell LE, Castagnino P (2008). Rac-dependent cyclin D1 gene expression regulated by cadherin- and integrin-mediated adhesion. *Journal of Cell Science*.

[199] Klein EA, Yin L, Kothapalli D (2009). Cell-cycle control by physiological matrix elasticity and in vivo tissue stiffening. *Current Biology*.

[200] Klein S, De Fougerolles AR, Blaikie P (2002). *α*5*β*1 integrin activates an NF-*κ*B-dependent program of gene expression important for angiogenesis and inflammation. *Molecular and Cellular Biology*.

[201] Reidy M, Zihlmann P, Hubbell JA, Hall H (2006). Activation of cell-survival transcription factor NF*κ*B in L1Ig6-stimulated endothelial cells. *Journal of Biomedical Materials Research Part A*.

[202] Scatena M, Almeida M, Chaisson ML, Fausto N, Nicosia RF, Giachelli CM (1998). NF-*κ*B mediates *α*v*β*3 integrin-induced endothelial cell survival. *Journal of Cell Biology*.

[203] Dormond O, Bezzi M, Mariotti A, Rüegg C (2002). Prostaglandin E2 promotes integrin *α*v*β*3-dependent endothelial cell adhesion, Rac-activation, and spreading through cAMP/PKA-dependent signaling. *Journal of Biological Chemistry*.

[204] Boosani CS, Mannam AP, Cosgrove D (2007). Regulation of COX-2-mediated signaling by *α*3 type IV noncollagenous domain in tumor angiogenesis. *Blood*.

[205] Kisseleva T, Song L, Vorontchikhina M, Feirt N, Kitajewski J, Schindler C (2006). NF-*κ*B regulation of endothelial cell function during LPS-induced toxemia and cancer. *Journal of Clinical Investigation*.

[206] Lahlou H, Sanguin-Gendreau V, Zuo D (2007). Mammary epithelial-specific disruption of the focal adhesion kinase blocks mammary tumor progression. *Proceedings of the National Academy of Sciences of the United States of America*.

[207] Pylayeva Y, Gillen KM, Gerald W, Beggs HE, Reichardt LF, Giancotti FG (2009). Ras- and PI3K-dependent breast tumorigenesis in mice and humans requires focal adhesion kinase signaling. *Journal of Clinical Investigation*.

[208] Samanna V, Wei H, Ego-Osuala D, Chellaiah MA (2006). Alpha-V-dependent outside-in signaling is required for the regulation of CD44 surface expression, MMP-2 secretion, and cell migration by osteopontin in human melanoma cells. *Experimental Cell Research*.

[209] Zutter MM, Santoro SA, Staatz WD, Tsung YL (1995). Re-expression of the *α*2*β*1 integrin abrogates the malignant phenotype of breast carcinoma cells. *Proceedings of the National Academy of Sciences of the United States of America*.

[210] Kren A, Baeriswyl V, Lehembre F (2007). Increased tumor cell dissemination and cellular senescence in the absence of *β*1-integrin function. *EMBO Journal*.

[211] Zhao H, Ross FP, Teitelbaum SL (2005). Unoccupied *α*v*β*3 integrin regulates osteoclast apoptosis by transmitting a positive death signal. *Molecular Endocrinology*.

[212] Frisch SM, Screaton RA (2001). Anoikis mechanisms. *Current Opinion in Cell Biology*.

[213] Stupack DG, Teitz T, Potter MD (2006). Potentiation of neuroblastoma metastasis by loss of caspase-8. *Nature*.

[214] Desgrosellier JS, Barnes LA, Shields DJ (2009). An integrin *α*(v)*β*(3)-c-Src oncogenic unit promotes anchorage-independence and tumor progression. *Nature Medicine*.

[215] Matter ML, Ruoslahti E (2001). A signaling pathway from the *α*5*β*1 and *α*v*β*3 integrins that elevates bcl-2 transcription. *Journal of Biological Chemistry*.

[216] Aoudjit F, Vuori K (2001). Matrix attachment regulates Fas-induced apoptosis in endothelial cells: a role for c-Flip and implications for anoikis. *Journal of Cell Biology*.

[217] Bao W, Strömblad S (2004). Integrin *α*v-mediated inactivation of p53 controls a MEK1-dependent melanoma cell survival pathway in three-dimensional collagen. *Journal of Cell Biology*.

[218] Alavi A, Hood JD, Frausto R, Stupack DG, Cheresh DA (2003). Role of Raf in vascular protection from distinct apoptotic stimuli. *Science*.

[219] Ruoslahti E (2002). Specialization of tumour vasculature. *Nature Reviews Cancer*.

[220] Alam N, Goel HL, Zarif MJ (2007). The integrin—growth factor receptor duet. *Journal of Cellular Physiology*.

[221] Serini G, Napione L, Arese M, Bussolino F (2008). Besides adhesion: new perspectives of integrin functions in angiogenesis. *Cardiovascular Research*.

[222] Wijelath ES, Rahman S, Namekata M (2006). Heparin-II domain of fibronectin is a vascular endothelial growth factor-binding domain: enhancement of VEGF biological activity by a singular growth factor/matrix protein synergism. *Circulation Research*.

[223] Zhu CQ, Popova SN, Brown ERS (2007). Integrin *α*11 regulates IGF2 expression in fibroblasts to enhance tumorigenicity of human non-small-cell lung cancer cells. *Proceedings of the National Academy of Sciences of the United States of America*.

[224] Orecchia A, Lacal PM, Schietroma C, Morea V, Zambruno G, Failla CM (2003). Vascular endothelial growth factor receptor-1 is deposited in the extracellular matrix by endothelial cells and is a ligand for the *α*5*β*1 integrin. *Journal of Cell Science*.

[225] Kajiya K, Hirakawa S, Ma B, Drinnenberg I, Detmar M (2005). Hepatocyte growth factor promotes lymphatic vessel formation and function. *EMBO Journal*.

[226] Murakami M, Elfenbein A, Simons M (2008). Non-canonical fibroblast growth factor signalling in angiogenesis. *Cardiovascular Research*.

[227] Camenisch G, Pisabarro MT, Sherman D (2002). ANGPTL3 stimulates endothelial cell adhesion and migration via integrin *α*v*β*3 and induces blood vessel formation in vivo. *Journal of Biological Chemistry*.

[228] Dallabrida SM, Ismail N, Oberle JR, Himes BE, Rupnick MA (2005). Angiopoietin-1 promotes cardiac and skeletal myocyte survival through integrins. *Circulation research*.

[229] Finger EC, Giaccia AJ (2010). Hypoxia, inflammation, and the tumor microenvironment in metastatic disease. *Cancer and Metastasis Reviews*.

[230] Billioux A, Modlich U, Bicknell R, Alison M (2007). Angiogenesis. *The Cancer Handbook*.

[231] Carmeliet P (2003). Angiogenesis in health and disease. *Nature Medicine*.

[232] Folkman J, Watson K, Ingber D, Hanahan D (1989). Induction of angiogenesis during the transition from hyperplasia to neoplasia. *Nature*.

[233] Weidner N, Semple JP, Welch WR, Folkman J (1991). Tumor angiogenesis and metastasis—correlation in invasive breast carcinoma. *New England Journal of Medicine*.

[234] Kandel J, Bossy-Wetzel E, Radvanyi F, Klagsbrun M, Folkman J, Hanahan D (1991). Neovascularization is associated with a switch to the export of bFGF in the multistep development of fibrosarcoma. *Cell*.

[235] Lin EY, Pollard JW (2007). Tumor-associated macrophages press the angiogenic switch in breast cancer. *Cancer Research*.

[236] Schmid MC, Varner JA (2007). Myeloid cell trafficking and tumor angiogenesis. *Cancer Letters*.

[237] Ferrara N, Gerber HP, LeCouter J (2003). The biology of VEGF and its receptors. *Nature Medicine*.

[238] Keeley EC, Mehrad B, Strieter RM (2011). Chemokines as mediators of tumor angiogenesis and neovascularization. *Experimental Cell Research*.

[239] Hong KH, Ryu J, Han KH (2005). Monocyte chemoattractant protein-1-induced angiogenesis is mediated by vascular endothelial growth factor-A. *Blood*.

[240] Niu J, Azfer A, Zhelyabovska O, Fatma S, Kolattukudy PE (2008). Monocyte chemotactic protein (MCP)-1 promotes angiogenesis via a novel transcription factor, MCP-1-induced protein (MCPIP). *Journal of Biological Chemistry*.

[241] Aplin AC, Fogel E, Nicosia RF (2010). MCP-1 promotes mural cell recruitment during angiogenesis in the aortic ring model. *Angiogenesis*.

[242] Iivanainen E, Kähäri VM, Heino J, Elenius K (2003). Endothelial cell-matrix interactions. *Microscopy Research and Technique*.

[243] Rundhaug JE (2005). Matrix metalloproteinases and angiogenesis. *Journal of Cellular and Molecular Medicine*.

[244] Murphy G, Nagase H (2011). Localizing matrix metalloproteinase activities in the pericellular environment. *FEBS Journal*.

[245] Ahn GO, Brown JM (2008). Matrix metalloproteinase-9 is required for tumor vasculogenesis but not for angiogenesis: role of bone marrow-derived myelomonocytic cells. *Cancer Cell*.

[246] Eilken HM, Adams RH (2010). Dynamics of endothelial cell behavior in sprouting angiogenesis. *Current Opinion in Cell Biology*.

[247] Risau W (1997). Mechanisms of angiogenesis. *Nature*.

[248] Folkman J (2002). Looking for a good endothelial address. *Cancer Cell*.

[249] Asahara T, Murohara T, Sullivan A (1997). Isolation of putative progenitor endothelial cells for angiogenesis. *Science*.

[250] Brellier F, Tucker RP, Chiquet-Ehrismann R (2009). Tenascins and their implications in diseases and tissue mechanics. *Scandinavian Journal of Medicine and Science in Sports*.

[251] Kaspar M, Zardi L, Neri D (2006). Fibronectin as target for tumor therapy. *International Journal of Cancer*.

[252] Midulla M, Verma R, Pignatelli M, Ritter MA, Courtenay-Luck NS, George AJT (2000). Source of oncofetal ED-B-containing fibronectin: implications of production of both tumor and endothelial cells. *Cancer Research*.

[253] Midwood KS, Orend G (2009). The role of tenascin-C in tissue injury and tumorigenesis. *Journal of Cell Communication and Signaling*.

[254] Degen M, Brellier F, Schenk S (2008). Tenascin-W, a new marker of cancer stroma, is elevated in sera of colon and breast cancer patients. *International Journal of Cancer*.

[255] Martina E, Chiquet-Ehrismann R, Brellier F (2010). Tenascin-W: an extracellular matrix protein associated with osteogenesis and cancer. *International Journal of Biochemistry and Cell Biology*.

[256] Döme B, Hendrix MJC, Paku S, Tóvári J, Tímár J (2007). Alternative vascularization mechanisms in cancer: pathology and therapeutic implications. *American Journal of Pathology*.

[257] Hillen F, Griffioen AW (2007). Tumour vascularization: sprouting angiogenesis and beyond. *Cancer and Metastasis Reviews*.

[258] Lyden D, Hattori K, Dias S (2001). Impaired recruitment of bone-marrow-derived endothelial and hematopoietic precursor cells blocks tumor angiogenesis and growth. *Nature Medicine*.

[259] Ribatti D (2004). The involvement of endothelial progenitor cells in tumor angiogenesis. *Journal of Cellular and Molecular Medicine*.

[260] Reyes M, Dudek A, Jahagirdar B, Koodie L, Marker PH, Verfaillie CM (2002). Origin of endothelial progenitors in human postnatal bone marrow. *Journal of Clinical Investigation*.

[261] Iruela-Arispe ML, Davis GE (2009). Cellular and molecular mechanisms of vascular lumen formation. *Developmental Cell*.

[262] Strilić B, Kučera T, Eglinger J (2009). The molecular basis of vascular lumen formation in the developing mouse aorta. *Developmental Cell*.

[263] Ghajar CM, George SC, Putnam AJ (2008). Matrix metalloproteinase control of capillary morphogenesis. *Critical Reviews in Eukaryotic Gene Expression*.

[264] Hildenbrand R, Allgayer H, Marx A, Stroebel P (2010). Modulators of the urokinase-type plasminogen activation system for cancer. *Expert Opinion on Investigational Drugs*.

[265] Bougatef F, Quemener C, Kellouche S (2009). EMMPRIN promotes angiogenesis through hypoxia-inducible factor-2*α*-mediated regulation of soluble VEGF isoforms and their receptor VEGFR-2. *Blood*.

[266] Brooks PC, Strömblad S, Sanders LC (1996). Localization of matrix metalloproteinase MMP-2 to the surface of invasive cells by interaction with integrin *α*v*β*3. *Cell*.

[267] Djonov V, Schmid M, Tschanz SA, Burri PH (2000). Intussusceptive angiogenesis. Its role in embryonic vascular network formation. *Circulation Research*.

[268] Kurz H, Burri PH, Djonov VG (2003). Angiogenesis and vascular remodeling by intussusception: from form to function. *News in Physiological Sciences*.

[269] Makanya AN, Hlushchuk R, Djonov VG (2009). Intussusceptive angiogenesis and its role in vascular morphogenesis, patterning, and remodeling. *Angiogenesis*.

[270] Brat DJ, Van Meir EG (2001). Glomeruloid microvascular proliferation orchestrated by VPF/VEGF: a new world of angiogenesis research. *American Journal of Pathology*.

[271] Straume O, Chappuis PO, Salvesen HB (2002). Prognostic importance of glomeruloid microvascular proliferation indicates an aggressive angiogenic phenotype in human cancers. *Cancer Research*.

[272] Holash J, Maisonpierre PC, Compton D (1999). Vessel cooption, regression, and growth in tumors mediated by angiopoietins and VEGF. *Science*.

[273] Döme B, Paku S, Somlai B, Timar J (2002). Vascularization of cutaneous melanoma involves vessel co-option and has clinical significance. *Journal of Pathology*.

[274] Scharpfenecker M, Fiedler U, Reiss Y, Augustin HG (2005). The Tie-2 ligand angiopoietin-2 destabilizes quiescent endothelium through an internal autocrine loop mechanism. *Journal of Cell Science*.

[275] Benjamin LE, Golijanin D, Itin A, Pode D, Keshet E (1999). Selective ablation of immature blood vessels in established human tumors follows vascular endothelial growth factor withdrawal. *Journal of Clinical Investigation*.

[276] Maniotis AJ, Folberg R, Hess A (1999). Vascular channel formation by human melanoma cells in vivo and in vitro: vasculogenic mimicry. *American Journal of Pathology*.

[277] Folberg R, Maniotis AJ (2004). Vasculogenic mimicry. *Acta Pathologica, Microbiologica. et Immunologica Scandinavica*.

[278] Pötgens AJG, Van Altena MC, Lubsen NH, Ruiter DJ, De Waal RMW (1996). Analysis of the tumor vasculature and metastatic behavior of xenografts of human melanoma cell lines transfected with vascular permeability factor. *American Journal of Pathology*.

[279] Clarijs R, Otte-Höller I, Ruiter DJ, De Waal RMW (2002). Presence of a fluid-conducting meshwork in xenografted cutaneous and primary human uveal melanoma. *Investigative Ophthalmology and Visual Science*.

[280] Kučera T, Lammert E (2009). Ancestral vascular tube formation and its adoption by tumors. *Biological Chemistry*.

[281] Ruf W, Seftor EA, Petrovan RJ (2003). Differential role of tissue factor pathway inhibitors 1 and 2 in melanoma vasculogenic mimicry. *Cancer Research*.

[282] Anand S, Majeti BK, Acevedo LM (2010). MicroRNA-132-mediated loss of p120RasGAP activates the endothelium to facilitate pathological angiogenesis. *Nature Medicine*.

[283] Anand S, Cheresh DA (2011). MicroRNA-mediated regulation of the angiogenic switch. *Current Opinion in Hematology*.

[284] Bellon G, Martiny L, Robinet A (2004). Matrix metalloproteinases and matrikines in angiogenesis. *Critical Reviews in Oncology/Hematology*.

[285] Nyberg P, Xie L, Kalluri R (2005). Endogenous inhibitors of angiogenesis. *Cancer Research*.

[286] Shimaoka M, Springer TA (2003). Therapeutic antagonists and conformational regulation of integrin function. *Nature Reviews Drug Discovery*.

[287] Eble JA, Haier J (2006). Integrins in cancer treatment. *Current Cancer Drug Targets*.

[288] Short SM, Derrien A, Narsimhan RP, Lawler J, Ingber DE, Zetter BR (2005). Inhibition of endothelial cell migration by thrombospondin-1 type-1 repeats is mediated by *β*1 integrins. *Journal of Cell Biology*.

[289] Zhang X, Lawler J (2007). Thrombospondin-based antiangiogenic therapy. *Microvascular Research*.

[290] Colorado PC, Torre A, Kamphaus G (2000). Anti-angiogenic cues from vascular basement membrane collagen. *Cancer Research*.

[291] Nyberg P, Xie L, Sugimoto H (2008). Characterization of the anti-angiogenic properties of arresten, an *α*1*β*1 integrin-dependent collagen-derived tumor suppressor. *Experimental Cell Research*.

[292] Woodall BP, Nyström A, Iozzo RA (2008). Integrin *α*2*β*1 is the required receptor for endorepellin angiostatic activity. *Journal of Biological Chemistry*.

[293] Mongiat M, Sweeney SM, San Antonio JD, Fu J, Iozzo RV (2003). Endorepellin, a novel inhibitor of angiogenesis derived from the C terminus of perlecan. *Journal of Biological Chemistry*.

[294] Marcinkiewicz C, Weinreb PH, Calvete JJ (2003). Obtu-statin: a potent selective inhibitor of *α*1*β*1 integrin in vitro and angiogenesis in vivo. *Cancer Research*.

[295] Brown MC, Staniszewska I, Del Valle L, Tuszynski GP, Marcinkiewicz C (2008). Angiostatic activity of obtustatin as *α*1*β*1 integrin inhibitor in experimental melanoma growth. *International Journal of Cancer*.

[296] Eble JA, Beermann B, Hinz H-J, Schmidt-Hederich A (2001). *α*2*β*1 integrin is not recognized by rhodocytin but is the specific, high affinity target of rhodocetin, an RGD-independent disintegrin and potent inhibitor of cell adhesion to collagen. *Journal of Biological Chemistry*.

[297] Eble JA, Niland S, Dennes A, Schmidt-Hederich A, Bruckner P, Brunner G (2002). Rhodocetin antagonizes stromal tumor invasion in vitro and other *α*2*β*1 integrin-mediated cell functions. *Matrix Biology*.

[298] Zhou J, Rothman VL, Sargiannidou I (2004). Cloning and characterization of angiocidin, a tumor cell binding protein for thrombospondin-1. *Journal of Cellular Biochemistry*.

[299] Sabherwal Y, Rothman VL, Dimitrov S (2006). Integrin*α*2*β*1 mediates the anti-angiogenic and anti-tumor activities of angiocidin, a novel tumor-associated protein. *Experimental Cell Research*.

[300] Pandey RC, Toussaint MW, McGuire JC, Thomas MC (1989). Maggiemycin and anhydromaggiemycin: two novel anthracyclinone antitumor antibiotics—isolation, structures, partial synthesis and biological properties. *Journal of Antibiotics*.

[301] Käpylä J, Pentikäinen OT, Nyrönen T (2007). Small molecule designed to target metal binding site in the *α*2I domain inhibits integrin function. *Journal of Medicinal Chemistry*.

[302] Nissinen L, Pentikäinen OT, Jouppila A (2010). A small-molecule inhibitor of integrin *α*2*β*1 introduces a new strategy for antithrombotic therapy. *Thrombosis and Haemostasis*.

[303] Funahashi Y, Sugi NH, Semba T (2002). Sulfonamide derivative, E7820, is a unique angiogenesis inhibitor suppressing an expression of integrin *α*2 subunit on endothelium. *Cancer Research*.

[304] O’Reilly MS, Boehm T, Shing Y (1997). Endostatin: an endogenous inhibitor of angiogenesis and tumor growth. *Cell*.

[305] Herbst RS, Hess KR, Tran HT (2002). Phase I study of recombinant human endostatin in patients with advanced solid tumors. *Journal of Clinical Oncology*.

[306] Dixelius J, Larsson H, Sasaki T (2000). Endostatin-induced tyrosine kinase signaling through the Shb adaptor protein regulates endothelial cell apoptosis. *Blood*.

[307] Karumanchi SA, Jha V, Ramchandran R (2001). Cell surface glypicans are low-affinity endostatin receptors. *Molecular Cell*.

[308] Wickström SA, Alitalo K, Keski-Oja J (2004). An endostatin-derived peptide interacts with integrins and regulates actin cytoskeleton and migration of endothelial cells. *Journal of Biological Chemistry*.

[309] Cianfrocca ME, Kimmel KA, Gallo J (2006). Phase 1 trial of the antiangiogenic peptide ATN-161 (Ac-PHSCN-NH 2), a beta integrin antagonist, in patients with solid tumours. *British Journal of Cancer*.

[310] Mould AP, Burrows L, Humphries MJ (1998). Identification of amino acid residues that form part of the ligand- binding pocket of integrin *α*5*β*1. *Journal of Biological Chemistry*.

[311] Marinelli L, Meyer A, Heckmann D, Lavecchia A, Novellino E, Kessler H (2005). Ligand binding analysis for human *α*5*β*1 integrin: strategies for designing new *α*5*β*1 integrin antagonists. *Journal of Medicinal Chemistry*.

[312] Umeda N, Kachi S, Akiyama H (2006). Suppression and regression of choroidal neovascularization by systemic administration of an *α*5*β*1 integrin antagonist. *Molecular Pharmacology*.

[313] Kuwada SK (2007). Volociximab, an angiogenesis-inhibiting chimeric monoclonal antibody. *Current Opinion in Molecular Therapeutics*.

[314] Wahl ML, Moser TL, Pizzo SV (2004). Angiostatin and anti-angiogenic therapy in human disease. *Recent Progress in Hormone Research*.

[315] Zhang D, Kaufman PL, Gao G, Saunders RA, Ma JX (2001). Intravitreal injection of plasminogen kringle 5, an endogenous angiogenic inhibitor, arrests retinal neovascularization in rats. *Diabetologia*.

[316] Ji WR, Castellino FJ, Chang Y (1998). Characterization of kringle domains of angiostatin as antagonists of endothelial cell migration, an important process in angiogenesis. *FASEB Journal*.

[317] Hamano Y, Kalluri R (2005). Tumstatin, the NC1 domain of *α*3 chain of type IV collagen, is an endogenous inhibitor of pathological angiogenesis and suppresses tumor growth. *Biochemical and Biophysical Research Communications*.

[318] Maeshima Y, Colorado PC, Kalluri R (2000). Two RGD-independent *α*(v)*β*3 integrin binding sites on tumstatin regulate distinct anti-tumor properties. *Journal of Biological Chemistry*.

[319] Floquet N, Pasco S, Ramont L (2004). The antitumor properties of the *α*3(IV)-(185–203) peptide from the NC1 domain of type IV collagen (tumstatin) are conformation-dependent. *Journal of Biological Chemistry*.

[320] Magnon C, Galaup A, Mullan B (2005). Canstatin acts on endothelial and tumor cells via mitochondrial damage initiated through interaction with *α*v*β*3 and *α*v*β*5 integrins. *Cancer Research*.

[321] Petitclerc E, Boutaud A, Prestayko A (2000). New functions for non-collagenous domains of human collagen type IV. Novel integrin ligands inhibiting angiogenesis and tumor growth in vivo. *Journal of Biological Chemistry*.

[322] Brooks PC, Silletti S, Von Schalscha TL, Friedlander M, Cheresh DA (1998). Disruption of angiogenesis by PEX, a noncatalytic metalloproteinase fragment with integrin binding activity. *Cell*.

[323] Bello L, Lucini V, Carrabba G (2001). Simultaneous inhibition of glioma angiogenesis, cell proliferation, and invasion by a naturally occurring fragment of human metalloproteinase-2. *Cancer Research*.

[324] Nam J-O, Jeong H-W, Lee B-H, Park R-W, Kim I-S (2005). Regulation of tumor angiogenesis by fastatin, the fourth FAS1 domain of *β*ig-h3, via *α*v*β*3 integrin. *Cancer Research*.

[325] Mas-Moruno C, Rechenmacher F, Kessler H (2010). Cilengitide: the first anti-angiogenic small molecule drug candidate. Design, synthesis and clinical evaluation. *Anti-Cancer Agents in Medicinal Chemistry*.

[326] Shannon KE, Keene JL, Settle SL (2004). Anti-metastatic properties of RGD-peptidomimetic agents S137 and S247. *Clinical and Experimental Metastasis*.

[327] Abdollahi A, Griggs DW, Zieher H (2005). Inhibition of *α*v*β*3 integrin survival signaling enhances antiangiogenic and antitumor effects of radiotherapy. *Clinical Cancer Research*.

[328] Tsopanoglou NE, Papaconstantinou ME, Flordellis CS, Maragoudakis ME (2004). On the mode of action of thrombin-induced angiogenesis: thrombin peptide, TP508, mediates effects in endothelial cells via *α* v*β*3 integrin. *Thrombosis and Haemostasis*.

[329] Meerovitch K, Bergeron F, Leblond L (2003). A novel RGD antagonist that targets both *α*v*β*3 and *α*5*β*1 induces apoptosis of angiogenic endothelial cells on type I collagen. *Vascular Pharmacology*.

[330] Kumar CC, Malkowski M, Yin Z (2001). Inhibition of angiogenesis and tumor growth by SCH 221153, a dual *α*v*β*3 and *α*v*β*5 integrin receptor antagonist. *Cancer Research*.

[331] Belvisi L, Riccioni T, Marcellini M (2005). Biological and molecular properties of a new *α*v*β*3/*α*v*β*5 integrin antagonist. *Molecular Cancer Therapeutics*.

[332] Minamiguchi K, Kumagai H, Masuda T, Kawada M, Ishizuka M, Takeuchi T (2001). Thiolutin, an inhibitor of huvec adhesion to vitronectin, reduces paxillin in huvecs and suppresses tumor cell-induced angiogenesis. *International Journal of Cancer*.

[333] Soldi R, Mitola S, Strasly M, Defilippi P, Tarone G, Bussolino F (1999). Role of *α*(v)*β*3 integrin in the activation of vascular endothelial growth factor receptor-2. *EMBO Journal*.

[334] Gutheil JC, Campbell TN, Pierce PR (2000). Targeted antiangiogenic therapy for cancer using vitaxin: a humanized monoclonal antibody to the integrin *α*(v)*β*3. *Clinical Cancer Research*.

[335] McNeel DG, Eickhoff J, Lee FT (2005). Phase I trial of a monoclonal antibody specific for *α* v*β*3 integrin (MEDI-522) in patients with advanced malignancies, including an assessment of effect on tumor perfusion. *Clinical Cancer Research*.

[336] Zhang D, Pier T, McNeel DG, Wilding G, Friedl A (2007). Effects of a monoclonal anti-*α*v*β*3 integrin antibody on blood vessels—a pharmacodynamic study. *Investigational New Drugs*.

[337] Hersey P, Sosman J, O’Day S (2010). A randomized phase 2 study of etaracizumab, a monoclonal antibody against integrin alpha(v)beta(3), + or - dacarbazine in patients with stage IV metastatic melanoma. *Cancer*.

[338] O'Day SJ, Pavlick AC, Albertini MR Clinical and pharmacologic evaluation of two dose levels of intetumumab (CNTO 95) in patients with melanoma or angiosarcoma.

[339] Varner JA, Nakada MT, Jordan RE, Coller BS (1999). Inhibition of angiogenesis and tumor growth by murine 7E3, the parent antibody of c7E3 Fab (abciximab; ReoPro). *Angiogenesis*.

[340] Nakada MT, Cao G, Sassoli PM, DeLisser HM (2006). c7E3 Fab inhibits human tumor angiogenesis in a SCID mouse human skin xenograft model. *Angiogenesis*.

[341] Mitjans F, Meyer T, Fittschen C (2000). In vivo therapy of malignant melanoma by means of antagonists of *α*v integrins. *International Journal of Cancer*.

[342] Reynolds AR, Hart IR, Watson AR (2009). Stimulation of tumor growth and angiogenesis by low concentrations of RGD-mimetic integrin inhibitors. *Nature Medicine*.

[343] Hanahan D (1998). A flanking attack on cancer. *Nature Medicine*.

[344] Jain RK (2005). Normalization of tumor vasculature: an emerging concept in antiangiogenic therapy. *Science*.

[345] Huang G, Chen L (2008). Tumor vasculature and microenvironment normalization: a possible mechanism of antiangiogenesis therapy. *Cancer Biotherapy and Radiopharmaceuticals*.

[346] Bergers G, Hanahan D (2008). Modes of resistance to anti-angiogenic therapy. *Nature Reviews Cancer*.

[347] Fraisl P, Mazzone M, Schmidt T, Carmeliet P (2009). Regulation of angiogenesis by oxygen and metabolism. *Developmental Cell*.

[348] Rapisarda A, Melillo G (2009). Role of the hypoxic tumor microenvironment in the resistance to anti-angiogenic therapies. *Drug Resistance Updates*.

[349] Lunt SJ, Chaudary N, Hill RP (2009). The tumor microenvironment and metastatic disease. *Clinical and Experimental Metastasis*.

[350] De Bock K, Cauwenberghs S, Carmeliet P (2010). Vessel abnormalization: another hallmark of cancer? Molecular mechanisms and therapeutic implications. *Current Opinion in Genetics and Development*.

[351] Reynolds AR (2010). Potential relevance of bell-shaped and u-shaped dose-responses for the therapeutic targeting of angiogenesis in cancer. *Dose-Response*.

[352] De S, Razorenova O, McCabe NP, O’Toole T, Qin J, Byzova TV (2005). VEGF—integrin interplay controls tumor growth and vascularization. *Proceedings of the National Academy of Sciences of the United States of America*.

[353] Mahabeleshwar GH, Chen J, Feng W, Somanath PR, Razorenova OV, Byzova TV (2008). Integrin affinity modulation in angiogenesis. *Cell Cycle*.

[354] Somanath PR, Ciocea A, Byzova TV (2009). Integrin and growth factor receptor alliance in angiogenesis. *Cell Biochemistry and Biophysics*.

[355] Somanath PR, Malinin NL, Byzova TV (2009). Cooperation between integrin *ανβ*3 and VEGFR2 in angiogenesis. *Angiogenesis*.

[356] Cretu A, Roth JM, Caunt M (2007). Disruption of endothelial cell interactions with the novel Hu177 cryptic collagen epitope inhibits angiogenesis. *Clinical Cancer Research*.

[357] Chen K, Chen X (2011). Integrin targeted delivery of chemotherapeutics. *Theranostics*.

[358] Wang Z, Chui WK, Ho PC (2010). Integrin targeted drug and gene delivery. *Expert Opinion on Drug Delivery*.

[359] Sugahara KN, Teesalu T, Prakash Karmali P (2010). Coadministration of a tumor-penetrating peptide enhances the efficacy of cancer drugs. *Science*.

[360] Strijkers GJ, Kluza E, Van Tilborg GAF (2010). Paramagnetic and fluorescent liposomes for target-specific imaging and therapy of tumor angiogenesis. *Angiogenesis*.

[361] Beer AJ, Kessler H, Wester HJ, Schwaiger M (2011). PET Imaging of Integrin alphaVbeta3 expression. *Theranostics*.

[362] Kiessling F, Gaetjens J, Palmowski M (2011). Application of molecular ultrasound for imaging integrin expression. *Theranostics*.

[363] Mery E, Jouve E, Guillermet S (2011). Intraoperative fluorescence imaging of peritoneal dissemination of ovarian carcinomas. A preclinical study. *Gynecologic Oncology*.

